# Longitudinal liver sampling in patients with chronic hepatitis B starting antiviral therapy reveals hepatotoxic CD8^+^ T cells

**DOI:** 10.1172/JCI158903

**Published:** 2023-01-03

**Authors:** Shirin Nkongolo, Deeqa Mahamed, Adrian Kuipery, Juan D. Sanchez Vasquez, Samuel C. Kim, Aman Mehrotra, Anjali Patel, Christine Hu, Ian McGilvray, Jordan J. Feld, Scott Fung, Diana Chen, Jeffrey J. Wallin, Anuj Gaggar, Harry L.A. Janssen, Adam J. Gehring

**Affiliations:** 1 Toronto Centre for Liver Disease, Toronto General Hospital Research Institute, University Health Network, Toronto, Ontario, Canada.; 2 Department of Immunology, University of Toronto, Toronto, Ontario, Canada.; 3 Gilead Sciences, Foster City, California, USA.; 4 Multi-Organ Transplant Program, Toronto General Hospital Research Institute, University Health Network, Toronto, Ontario, Canada.

**Keywords:** Immunology, Infectious disease, Cellular immune response, Hepatitis, T cells

## Abstract

Accumulation of activated immune cells results in nonspecific hepatocyte killing in chronic hepatitis B (CHB), leading to fibrosis and cirrhosis. This study aims to understand the underlying mechanisms in humans and to define whether these are driven by widespread activation or a subpopulation of immune cells. We enrolled CHB patients with active liver damage to receive antiviral therapy and performed longitudinal liver sampling using fine-needle aspiration to investigate mechanisms of CHB pathogenesis in the human liver. Single-cell sequencing of total liver cells revealed a distinct liver-resident, polyclonal CD8^+^ T cell population that was enriched at baseline and displayed a highly activated immune signature during liver damage. Cytokine combinations, identified by in silico prediction of ligand-receptor interaction, induced the activated phenotype in healthy liver CD8^+^ T cells, resulting in nonspecific Fas ligand–mediated killing of target cells. These results define a CD8^+^ T cell population in the human liver that can drive pathogenesis and a key pathway involved in their function in CHB patients.

## Graphical abstract

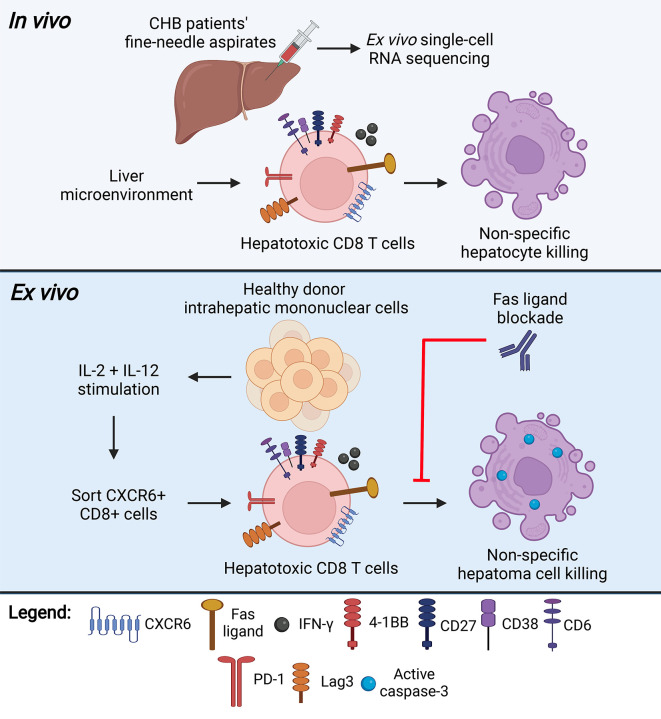

## Introduction

Nonspecific activation of memory CD8^+^ T cells is a common feature of acute and chronic human viral diseases (1–7). Bystander-activated CD8^+^ T cells respond in a TCR-independent manner and can contribute to protective immunity or mediate tissue damage through cytotoxic mechanisms (8, 9). Tissue damage mediated by nonspecific CD8^+^ T cell activation is not restricted to viral infections and has been reported in type 1 diabetes (10), graft-versus-host disease (11), celiac disease (12), and Lyme arthritis (13). Despite the critical role of CD8^+^ T cells in host protection, dysregulation of CD8^+^ T cell activation can drive immune-mediated pathogenesis, particularly in chronic diseases.

Persistent liver damage is the primary driver of disease progression in chronic HBV infection. Nonspecific liver injury leads to fibrosis, cirrhosis, and hepatocellular carcinoma, with about 820,000 HBV-related deaths per year (14–17). Mechanisms of liver damage have been delineated in animal models of acute HBV infection. Functional HBV-specific CD8^+^ T cells produce IFN-γ, which induces the chemokines CXCL9 and CXCL10 to coordinate recruitment of inflammatory immune cells and cause liver damage (18–21). In contrast to what occurs in acute infection models, HBV-specific CD8^+^ T cells are deleted in chronic hepatitis B (CHB) patients due to persistent antigen exposure. The remaining HBV-specific CD8^+^ T cells are exhausted (22–27), and their frequency inversely correlates with liver damage, indicating they are not the primary drivers of liver damage in CHB (6, 28). Therefore, the role of CD8^+^ T cells in nonspecific liver damage, mechanisms responsible for nonspecific hepatocyte killing, and the source of IFN-γ to initiate the inflammatory infiltrate in human chronic HBV infection remain poorly defined (6, 9).

A major obstacle to understanding CHB pathogenesis is the virus’ narrow host range and lack of chronic infection models that mimic decades of infection for studying pathogenic mechanisms in the liver. To overcome these obstacles, we used longitudinal liver fine-needle aspiration (FNA) (29, 30) of CHB patients with active liver damage who started antiviral therapy with the nucleotide analogue tenofovir alafenamide (TAF). TAF treatment inhibits viral replication and reduces liver damage, allowing us to quantify dynamic changes in the intrahepatic immunological landscape using single-cell RNA sequencing (scRNA-Seq). This approach identified a highly activated, tissue-resident CD8^+^ T cell population that caused Fas ligand–dependent (FasL-dependent) apoptosis in hepatoma cells, consistent with their role in liver damage, and expressed IFN-γ, which can drive the inflammatory infiltrate. This indicates that innate inflammatory cytokines can drive bystander CD8^+^ T cell activation to initiate the inflammatory process in CHB patients without a requirement for IFN-γ derived from HBV-specific T cells.

## Results

### Longitudinal liver FNAs were collected corresponding to response to antiviral therapy.

In this study, data are shown from 9 patients who, at the time of enrollment, had active CHB with elevated alanine aminotransferase (ALT) levels, mean ALT 8.9 × upper limit of normal (ULN) (range: 1.1–21.8 × ULN), and had started to receive 25 mg/d TAF (Supplemental Figure 1A; supplemental material available online with this article; https://doi.org/10.1172/JCI158903DS1). Baseline characteristics are summarized in Supplemental Table 1. After patients started TAF therapy, ALT levels decreased by a mean of 8.4-fold (up to 20.5-fold) by week 12 and by a mean of 11.0-fold (up to 21-fold) by week 24 (Supplemental Figure 1B). At week 24, 7 of 9 patients had normalized ALT, with all patients ranging between 0.4 and 1.2 × ULN. HBV DNA levels ranged between 2.73 × 10^5^ and 9.97 × 10^7^ IU/ml (mean: 3.09 × 10^7^ IU/ml) at screening, and these decreased below detection limit in 4 patients, with a maximum of 285 IU/ml and a mean of 47 IU/ml in all patients at week 24. Hepatitis B surface antigen (HBsAg) levels did not significantly decrease. Out of 4 HBeAg-positive patients at baseline, 1 underwent HBeAg seroconversion by week 12.

### scRNA-Seq of liver FNAs captures a comprehensive immunological picture of the human liver.

**Figure 1:**
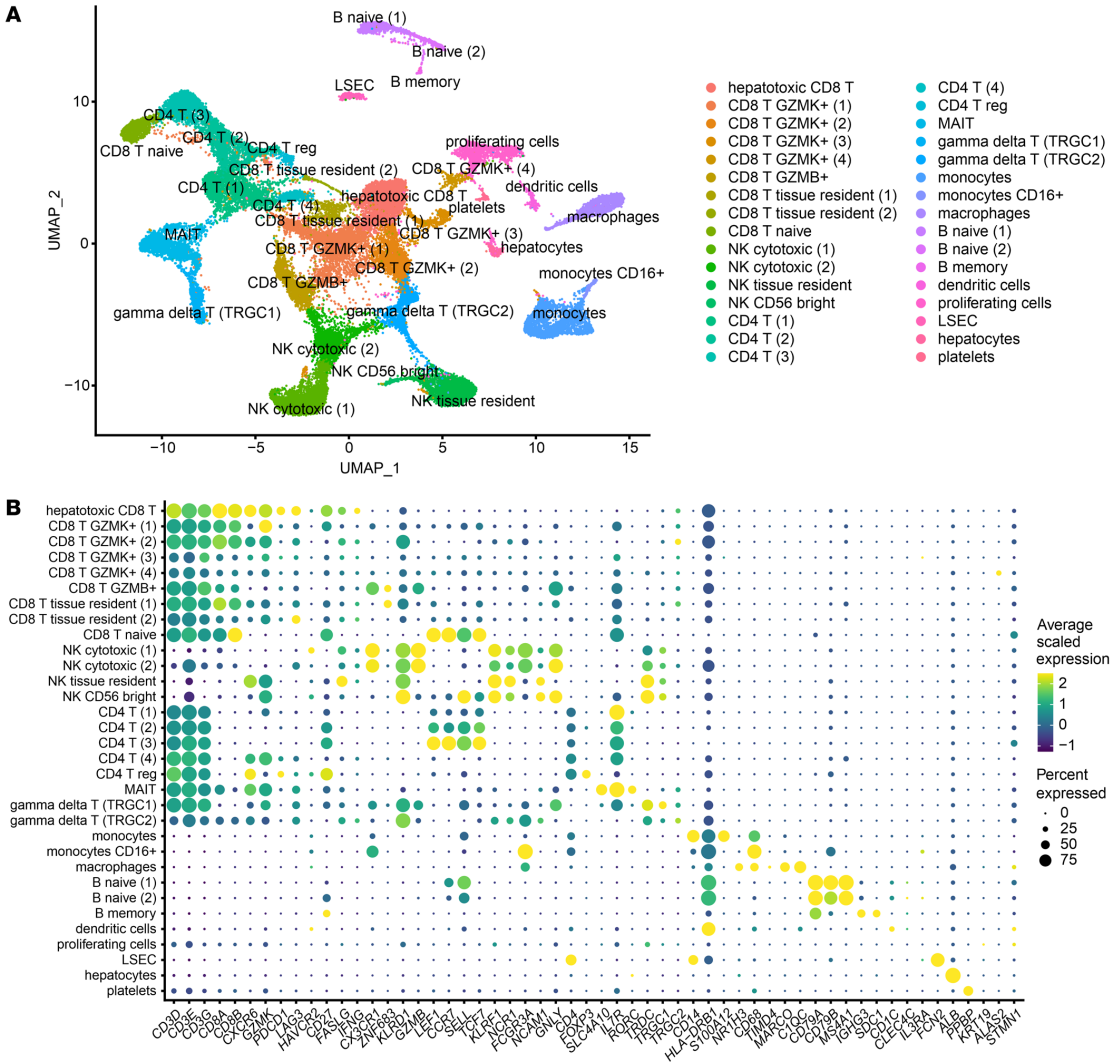
scRNA-Seq revealed 32 different populations. (**A**) Cells from all 5 patients at all 3 time points were filtered to exclude low-quality cells and doublets and were clustered and displayed in a UMAP plot. (**B**) Cell types were assigned to each cluster using selected marker genes. Whenever the dominant cell type of a cluster was unclear, we used differential gene expression testing and analysis of overexpressed signalling pathways in addition to the displayed marker genes.

The full cellular diversity of liver FNAs has not been mapped, and given the fragility of some cell types (hepatocytes) or adhesion to the parenchyma (macrophages, endothelial, and stellate cells), it was not obvious if we could consistently capture cellular diversity in serial FNAs to study changes in activation status by scRNA-Seq. scRNA-Seq data from 5 patients before and 12 and 24 weeks after starting TAF therapy were integrated following a pipeline to minimize batch effects and eliminate low-quality cells damaged during processing. 38,036 High-quality cells were analyzed and clustered into 32 distinct populations (Figure 1A). Cells from all clusters showed consistent distribution among the different patients (Supplemental Figure 2; numbers of cells from each cluster obtained from each patient are provided in Supplemental Table 2). These data demonstrate consistent capturing of diverse immune cell types through longitudinal FNA sampling of the HBV-infected liver.

**Figure 2:**
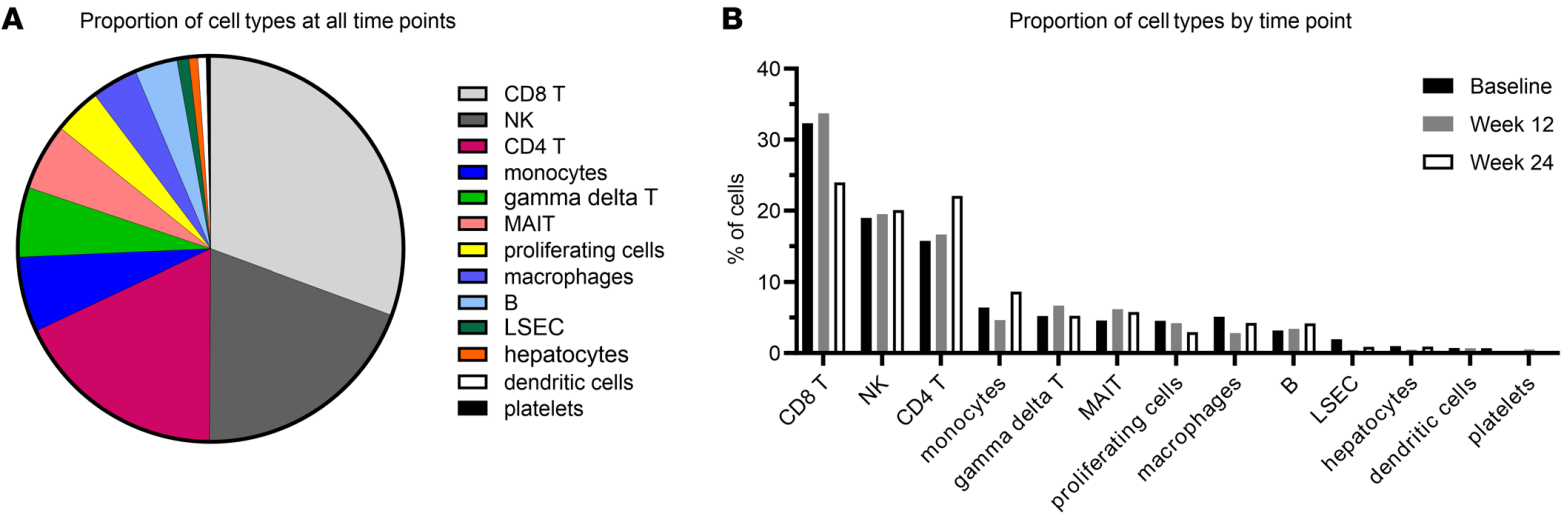
CD8^+^ T cells were the most abundant cell type according to scRNA-Seq. 38,036 High-quality cells from all 5 patients at all 3 time points were analyzed. (**A**) Distribution of cell types across all time points and (**B**) by time point. Note that due to the sampling method, there are limitations to capturing all cell types present in the liver (in particular, adherent cells); displayed frequencies are relative to all captured cells. CD8^+^ T cells are the predominant cell type at all time points, but their proportion decreases from baseline to week 24.

Cell types were annotated using key lineage and phenotypic marker genes (Figure 1B). Consistent with previous studies (29, 30), liver FNAs were dominated by immune cells that can be mobilized with aspiration, whereas parenchymal (hepatocytes, endothelial cells) or tightly bound cells (macrophages) were present but underrepresented (Figure 2A). CD8^+^ T cells were most frequent (30.6% of all cells), followed by NK cells (19.5%) and CD4^+^ T cells (17.9%). Less frequent were monocytes (6.3%), γδ T cells (5.9%), mucosal-associated invariant T (MAIT) cells (5.6%), macrophages (3.9%), and B cells (3.6%). The 10x Genomics sequencing approach did not capture significant numbers of granulocytes. Dendritic cells and nonimmune populations — liver sinusoidal endothelial cells (LSECs), hepatocytes, and platelets — each represented less than 1% of all cells. Hepatocytes were largely filtered out as low-quality cells and accounted for 0.8% of high-quality cells in our data set, most likely due to limited yield with aspiration alone rather than biopsy, low stability during sample processing, and strict mitochondrial RNA content cut-off of 10%. One cluster of proliferating cells did not display a distinct cell type, but showed upregulation of cell cycle–associated genes and pathways; it accounted for 4% of all cells.

We determined changes in immune cell composition in the liver over time, as TAF therapy suppressed viral load and normalized ALT. The frequency of CD8^+^ T cells was highest at baseline, during active liver damage, and decreased over time (32.3% of all cells at baseline and 24.0% at week 24; paired *t* test: *P* = 0.06) (Figure 2B). In contrast, the proportion of CD4^+^ T cells increased over time (15.7% at baseline and 22.1% at week 24; paired *t* test: *P* = 0.18). All other cell types showed less change over time, suggesting the enrichment of CD8^+^ T cells at baseline may be physiologically relevant, given their known role in liver damage.

Overall, scRNA-Seq of longitudinal FNAs did not effectively represent parenchymal cells, but consistently captured immune cells, allowing us to investigate dynamic changes in transcriptional profiles as ALT levels declined under antiviral treatment.

### Hepatotoxic CD8^+^ T cells have a unique activated transcriptomic signature.

**Figure 3:**
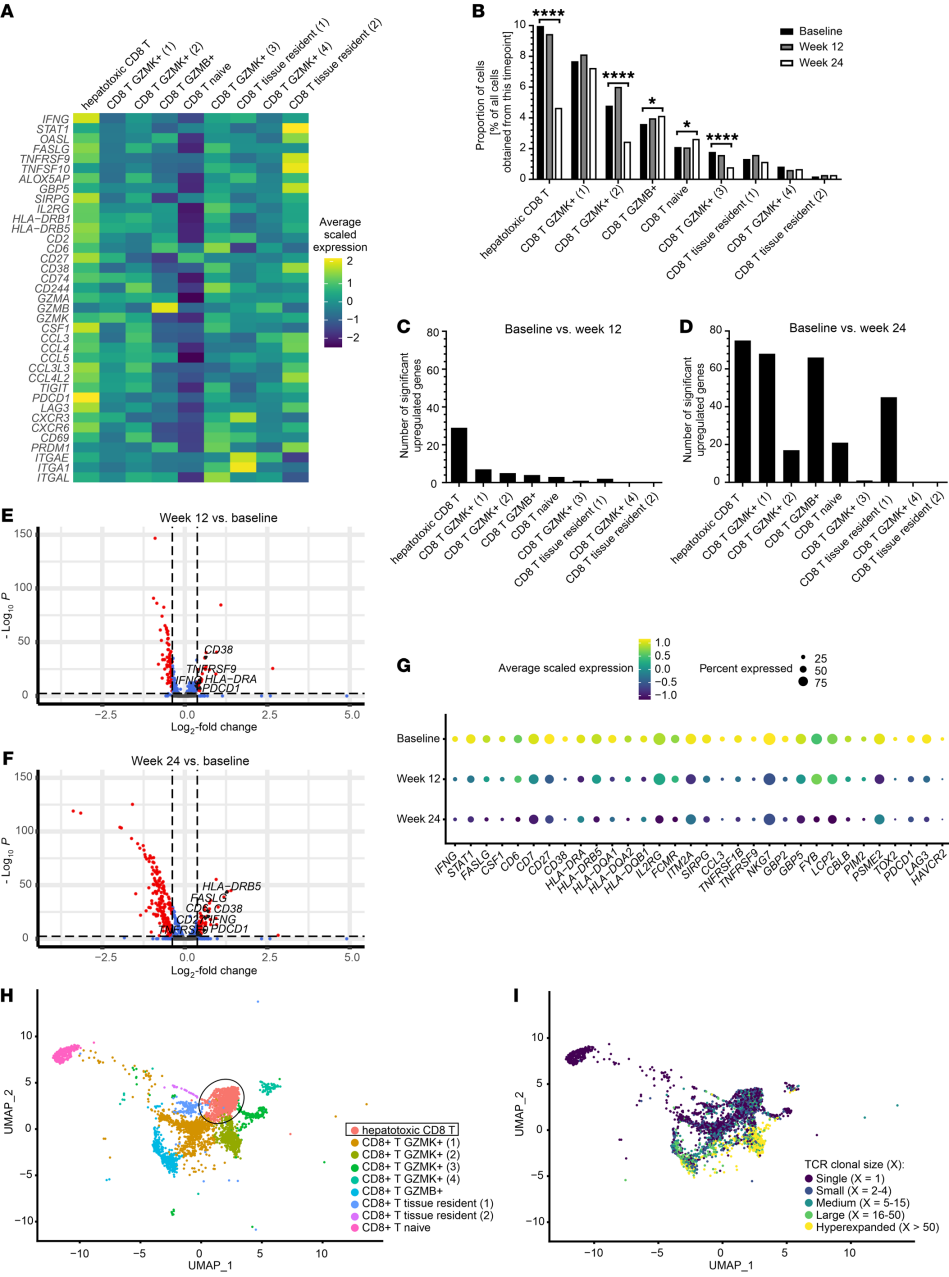
Hepatotoxic CD8^+^ T cells are a unique inflammatory and polyclonal CD8^+^ T cell population that downregulates immune-related genes as ALT levels decline. Data pooled from all 5 patients. (**A**) Heatmap displaying a unique activated immunological signature of hepatotoxic CD8^+^ T cells at baseline. (**B**) Proportions of cells in the CD8^+^ T cell clusters out of all cells obtained at the respective time points. *P < 0.05; ****P < 0.0001, 2-sided *z* test to test for significant enrichment at baseline or week 24. 3,144 Cells make up the hepatotoxic CD8^+^ T cell population. (**C** and **D**) Numbers of differentially upregulated genes in each CD8^+^ T cell cluster when comparing baseline and week 12 (**C**) and baseline and week 24 (**D**). (**E** and **F**) Volcano plot showing genes that are differentially expressed over time by 12 weeks (**E**) and by 24 weeks (**F**) in the hepatotoxic CD8^+^ T cell population. Thresholds: *P* < 0.005 and fold change ≥ 1.3. Genes upregulated at baseline are shown to the right side of each plot. Genes downregulated at baseline are mainly ribosomal and mitochondrial genes. (**G**) Expression of immune-related genes in hepatotoxic CD8^+^ T cells from baseline to week 24. (**H**) UMAP plot of CD8^+^ T cell clusters at the time of active liver damage. (**I**) UMAP plot in (**H**) overlaid with the respective clonal sizes of TCR clonotypes.

Because CD8^+^ T cells primarily mediate HBV-related liver damage in animal models, were the most abundant cell type in our scRNA-Seq dataset, and were the only cell type that decreased between baseline and week 24, they were analyzed in greater detail. We identified 9 distinct CD8^+^ T cell populations (Figure 3A). One population showed a tissue-resident (*CXCR6*; *CD69*), activated signature, including high expression of effector molecules (*IFNG*, *FASLG*, granzyme A [*GZMA*], granzyme K [*GZMK*]), chemokines (*CCL3*, *CCL4*, *CCL5*), activation markers (*TNFRSF9* [CD137/4-1BB], *CD38*, *HLA-DR*, *CD27*, *CD74*), and exhaustion markers (*PDCD1* [PD-1], *LAG3*, *TIGIT*). Given this highly activated phenotype and expression of effector molecules associated with liver damage, we termed this population hepatotoxic CD8^+^ T cells.

Hepatotoxic CD8^+^ T cells were the largest population and comprised 3,144 cells from all patients combined, representing 27% of cells in CD8^+^ T cell clusters (8.3% of all cells). This population, along with 2 other populations (CD8^+^ T GZMK^+^ 2 and 3), was significantly enriched at baseline compared with week 24 (Figure 3B). By week 24, when ALT levels had largely normalized, hepatotoxic CD8^+^ T cells had contracted by 53%. As a measure of activation changes, we quantified significantly differentially expressed genes (DEGs) that were downregulated from baseline to weeks 12 and 24 after starting therapy. Hepatotoxic CD8^+^ T cells showed the greatest changes in DEGs as ALT declined, with 29 genes significantly decreased by week 12 (based on a Bonferroni’s corrected adjusted *P* < 0.005 and a fold change ≥ 1.3), when mean ALT had declined 8.4-fold. All other CD8^+^ T cell clusters had only 0 to 7 significantly downregulated DEGs (Figure 3C). The number of significantly downregulated DEGs in hepatotoxic CD8^+^ T cells increased to 75 when comparing baseline with week 24 (Figure 3D). By that time, other CD8^+^ T cell clusters also showed significant downregulation, with DEGs ranging between 0 and 68.

In total, 80 unique genes were significantly upregulated in hepatotoxic CD8^+^ T cells at baseline, during active liver damage, compared with the later time points (Figure 3, E and F). The largest proportion of these genes were immune related (*n* = 32), followed by mitochondrial and ribosomal genes (*n* = 14) and genes regulating transcription/translation (*n* = 8). In contrast, genes that were upregulated following decline of ALT levels at weeks 12 and 24 were overwhelmingly mitochondrial and ribosomal genes. The 32 immune-related genes upregulated during active liver damage in hepatotoxic CD8^+^ T cells decreased both in mean expression levels in the individual cells and in the percentages of transcript-positive cells (Figure 3G).

Further characterization using TCR clonotypes revealed both polyclonal and hyperexpanded CD8^+^ T cell populations (Figure 3, H and I). Naive CD8^+^ T cells — as expected — displayed only single clonotypes unique for each cell (clonal size of 1). In contrast, most cells in the CD8^+^ T GZMK^+^ 2 population were clonally expanded. Hepatotoxic CD8^+^ T cells were polyclonal, with the majority of cells (76%) either having a unique clonotype or a clonotype of clonal size of 4 or less in each sample, and 93% of cells having a clonotype of clonal size of 15 or less. We compared data from hepatotoxic CD8^+^ T cells to that in publicly available TCR sequences. When searching the Immune Epitope Database and Analysis Resource (IEDB; https://www.iedb.org/), available TCR data matched TCR sequences described as specific for common viruses such as Epstein-Barr virus (3.6% of cells in this cluster), coronavirus (0.5%), and influenza A virus (3.6%). This was in line with the polyclonality of this cluster.

Hepatotoxic CD8^+^ T cells stood out from all CD8^+^ T cell populations due to high expression of activation markers and effector molecules, especially at baseline. This was the largest of all CD8^+^ T cell populations with relative enrichment at the time of active liver damage, and it displayed a polyclonal TCR composition. It was the only CD8^+^ T cell population that showed significant downregulation of a substantial number of genes from baseline to week 12, corresponding to the main decrease in ALT levels. Under TAF therapy, the expression of immune-related activation genes decreased, including that of *IFNG* and *FASLG*. Therefore, hepatotoxic CD8^+^ T cells displayed all hallmarks of a population with the potential to drive inflammatory events leading to liver damage.

### The hepatotoxic CD8^+^ T cell population is validated at the protein level in liver FNAs.

Transcriptomic data were validated at the protein level in cryopreserved FNAs using flow cytometry. Markers and gating strategy are displayed in Supplemental Figure 3. Due to low numbers of cells recovered from cryopreserved FNAs, we focused on extracellular markers to minimize cell loss during sample staining. Longitudinal FNAs and matching PBMCs from 4 patients were analyzed. FNAs contained significantly more tissue-resident CXCR6^+^CD8^+^ T cells than PBMCs (FNA: mean 29.3% of CD8^+^ T cells were CXCR6^+^; PBMCs: mean 12.0%; Supplemental Figure 4A).

**Figure 4:**
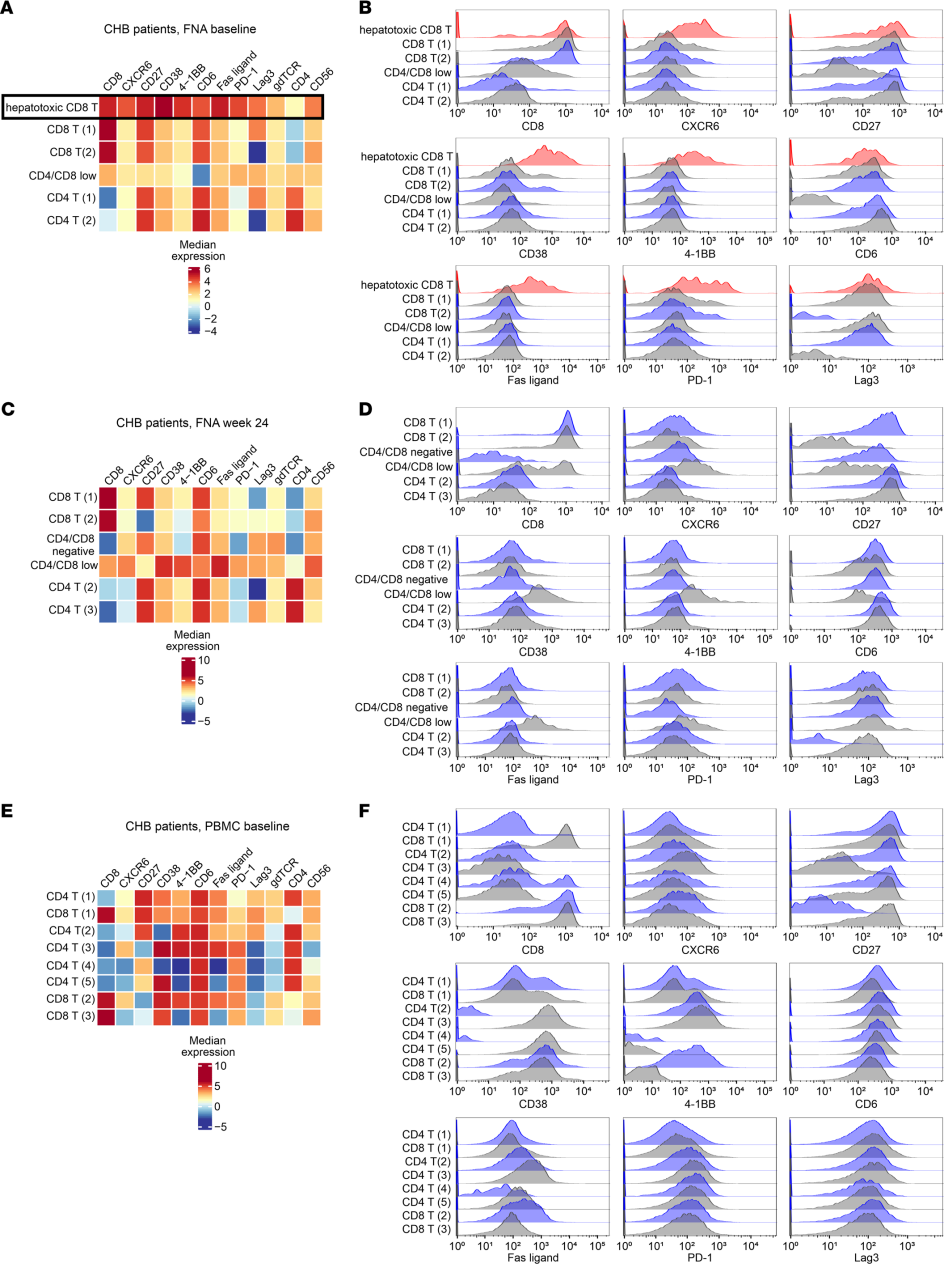
The hepatotoxic CD8^+^ T cell population can be identified on the protein level in FNAs from patients with active liver damage, but not after 24 weeks of TAF therapy and not in PBMCs. Using a multicolor flow cytometry panel for FNA samples from 4 CHB patients with ongoing liver damage (**A** and **B**) and as ALT levels had largely normalized (**C** and **D**) and corresponding PBMC samples from CHB patients with active liver damage (**E** and **F**), single, live CD3^+^ lymphocytes were selected and clustered. Heatmaps show median fluorescence intensity; histograms display the distribution of expression levels for CD8 and key hepatotoxic CD8^+^ T cell phenotypic markers for each cluster.

Using R packages flowCore and CATALYST, we clustered CD3^+^ cells from the different sample types (FNAs/PBMCs) from 4 additional patients at baseline or at week 24 (Figure 4). For FNAs stratified by time point, a resolution leading to 6 clusters was optimal, since additional clusters did not improve cluster stability. In baseline FNAs, 3 CD8^+^ T cell populations were identified: 2 CD4^+^ T cell populations and 1 population with low expression of CD4 and CD8 (Figure 4, A and B). One CD8^+^ T cell population had distinctly higher expression of CXCR6, CD38, 4-1BB, FasL, and PD-1 than all other clusters. This population highly expressed CD27 and CD6, corresponding to hepatotoxic CD8^+^ T cells defined by scRNA-Seq. This population was consistently identified at any resolution between 4 and 20 clusters.

In contrast with baseline samples, week 24 FNAs (Figure 4, C and D) displayed 2 clusters with high CD8 expression, but low or negative expression of most phenotypic markers defining hepatotoxic CD8^+^ T cells. One population (CD4/CD8 low) expressed both CD4 and CD8 at low-to-moderate levels and had high expression of CD38, 4-1BB, and FasL, but only low-to-moderate levels of the other markers. Consistent with the transcriptional data showing downregulation of immune-related genes at week 24, a clear hepatotoxic CD8^+^ T cell cluster could not be identified at the protein level. This held true for any clustering resolution between 4 and 20 clusters.

Finally, we determined whether a hepatotoxic CD8^+^ T cell population could be identified in matching PBMCs from the same patients at baseline. PBMC samples (Figure 4, E and F) needed to be clustered at a higher resolution to display more than one CD8^+^ T cell population. No cluster displayed high expression of CXCR6 or the combination of surface markers defining hepatotoxic CD8^+^ T cells (Figure 4E). Therefore, a distinct hepatotoxic CD8^+^ T cell population was identified at the protein level in CHB patient liver FNAs at the time of active liver damage, but not in liver FNAs after 24 weeks of TAF therapy or in matched PBMC samples. This indicates that the hepatotoxic CD8^+^ T cell population exists uniquely in the liver during ongoing liver damage.

### IL-2 and IL-12 induce hepatotoxic CD8^+^ T cells in intrahepatic mononuclear cells.

We anticipated that cytokines present in the inflammatory liver environment were responsible for hepatotoxic CD8^+^ T cell activation. Only low numbers of immune cells could be obtained from FNAs: each pass typically yielded 20,000 to 50,000 live cells, with 4 passes taken from each patient per time point. To conduct functional experiments, we took advantage of intrahepatic mononuclear cells (IHMCs) harvested from liver perfusion samples (Supplemental Figure 4). In contrast with FNAs, these provide higher cell numbers and originate from noninfected individuals with no significant liver inflammation or liver damage.

We recently defined the inflammatory profile that is associated with liver damage in CHB patients (31). Therefore, we aimed to define crucial factors to mimic the liver microenvironment and induce a population correlating to hepatotoxic CD8^+^ T cells in IHMCs. Using NicheNet, we predicted in silico factors responsible for upregulation of 2 key effector genes defining hepatotoxic CD8^+^ T cells, FasL and IFN-γ, at baseline. Table 1 shows the top 25 ligands with the highest predicted potentials for FasL and IFN-γ. Notably, there was a distinct overlap between the predicted ligands for upregulation of both genes.

In exploratory experiments for ex vivo verification, we selected cytokines with the highest predictive value for FasL upregulation that also appeared in the list for IFN-γ. We selected IL-2, IL-4, IL-10, IL-12p70 (corresponding to IL-12A and IL-12B), IL-15, IL-21, IL-27, TGF-β, TNF, IFN-α, and IFN-γ (Supplemental Figure 5, A and C) and some combinations thereof (Supplemental Figure 5, B and D) to stimulate IHMCs for 24 hours. FasL and IFN-γ upregulation in IHMC-derived CXCR6^+^CD8^+^ T cells was quantified by flow cytometry. The combination of IL-2 and IL-12 induced both proteins most effectively.

**Figure 5:**
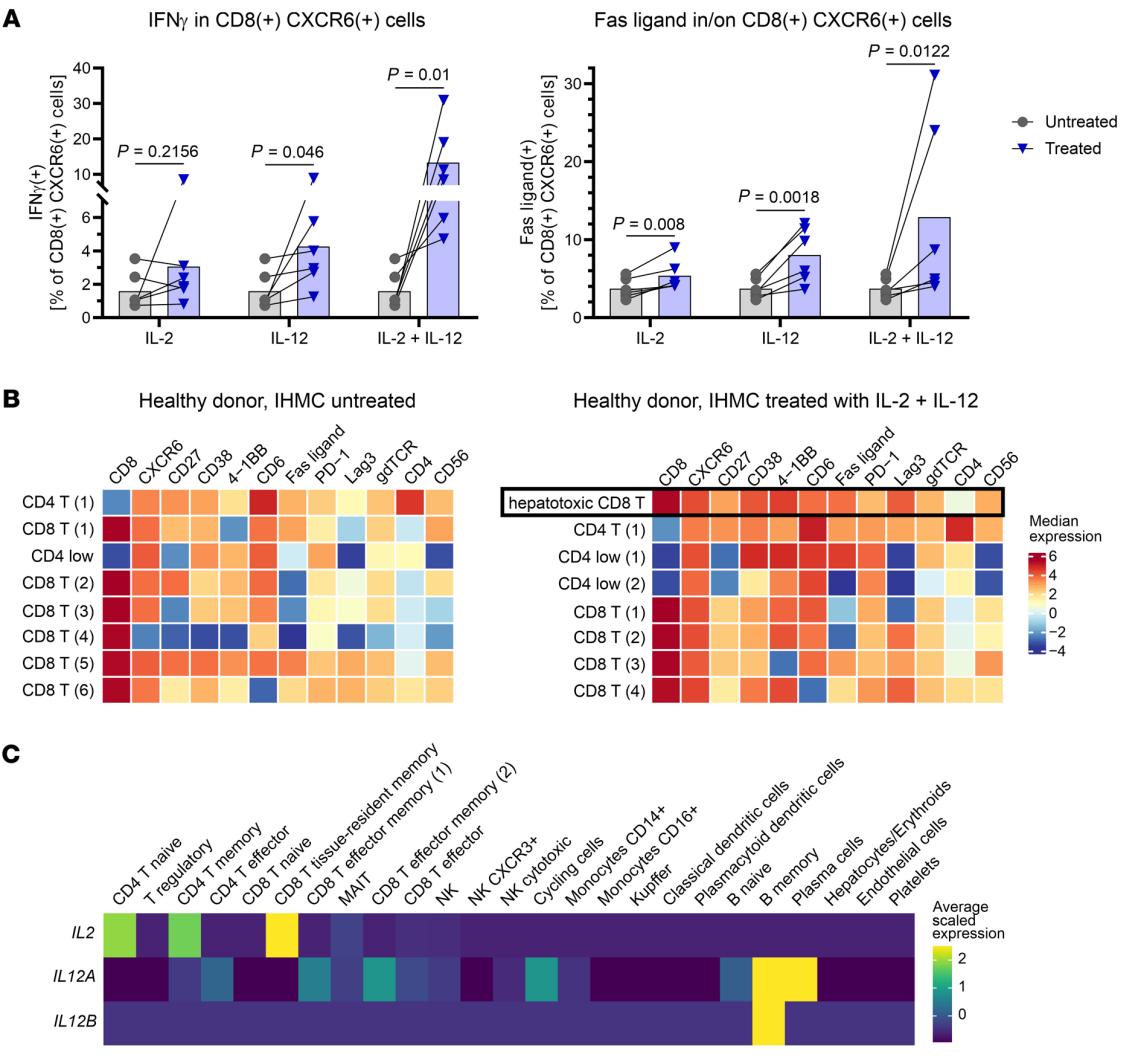
The combination of IL-2 and IL-12 induces hepatotoxic CD8^+^ T cells in healthy donor intrahepatic cells. (**A**) IHMCs from 6 healthy donors were treated with IL-2, IL-12, or IL-2 plus IL-12 for 24 hours before quantification of IFN-γ and FasL expression of CXCR6^+^CD8^+^ T cells by flow cytometry. Circles and triangles indicate individual donors; bars indicate mean values. Statistical significance was assessed by 2-tailed, ratio-paired *t* test. (**B**) A multicolor flow cytometry panel was used to analyze IHMCs from 6 healthy donors, with and without 24 hours of treatment with IL-2 plus IL-12. Single, live CD3^+^ lymphocytes were selected before clustering. The hepatotoxic CD8^+^ T cell population, corresponding to baseline FNA samples from CHB patients, was only found in the treated healthy donor IHMCs. Heatmaps show median expression. (**C**) Source of *IL2* and *IL12* in CHB patients’ livers at baseline. A targeted scRNA-Seq assay was used to enrich for cytokine genes. Both *IL2* and *IL12A/IL12B* could be detected.

Among CXCR6^+^CD8^+^ T cells, the combination of IL-2 and IL-12 increased IFN-γ^+^ cells from 1.6% to 13.4% and FasL^+^ cells from 3.7% to 12.9% (Figure 5A). In comparison, 19.8% of CXCR6^+^CD8^+^ T cells were FasL^+^, assessed by flow cytometry of baseline FNAs from CHB patients (IFN-γ as an intracellular cytokine was not assessed). We confirmed that the broader panel of hepatotoxic CD8^+^ T cell markers was induced on IHMCs by IL-2 plus IL-12 treatment for 24 hours (Figure 5B and Supplemental Figure 6; gating strategy in Supplemental Figure 3). Untreated IHMCs showed 6 CD8^+^ T cell populations (out of a total of 8 populations), none of which highly expressed all hepatotoxic CD8^+^ T cell markers. One CD8^+^ T cell population, CD8^+^ T 5, accounting for 11.3% of CD3^+^ lymphocytes, expressed hepatotoxic CD8^+^ T cell markers at a low-to-moderate level. In contrast, after IL-2 plus IL-12 treatment, a hepatotoxic CD8^+^ T cell population with medium-to-high expression of all markers of interest was identified, which made up 24.4% of T cells. These data indicate that exposure to IL-2 plus IL-12 can induce a CD8^+^ T cell population with a phenotype similar to the one observed in liver FNAs from CHB patients during active liver damage.

IL-2 and IL-12 were identified in the plasma of patients with active hepatitis (31). To determine the source of IL-2 and IL-12 in CHB patients at baseline, we used a targeted gene expression kit for enrichment of 1,056 genes that included all cytokines of interest. *IL2* was mainly expressed by CD4^+^ T cells and a tissue-resident memory CD8^+^ T cell population. *IL12* (*IL12A* + *IL12B*) was expressed by B cells (Figure 5C). In the scRNA-Seq data from CHB patients, CD4^+^ T cells, CD8^+^ T cells, and B cells were therefore identified as likely sources of the cytokines confirmed to induce the hepatotoxic CD8^+^ T cell phenotype.

### IHMC-derived hepatotoxic CD8^+^ T cells can induce apoptosis in human hepatoma cells in a FasL-dependent manner.

**Figure 6:**
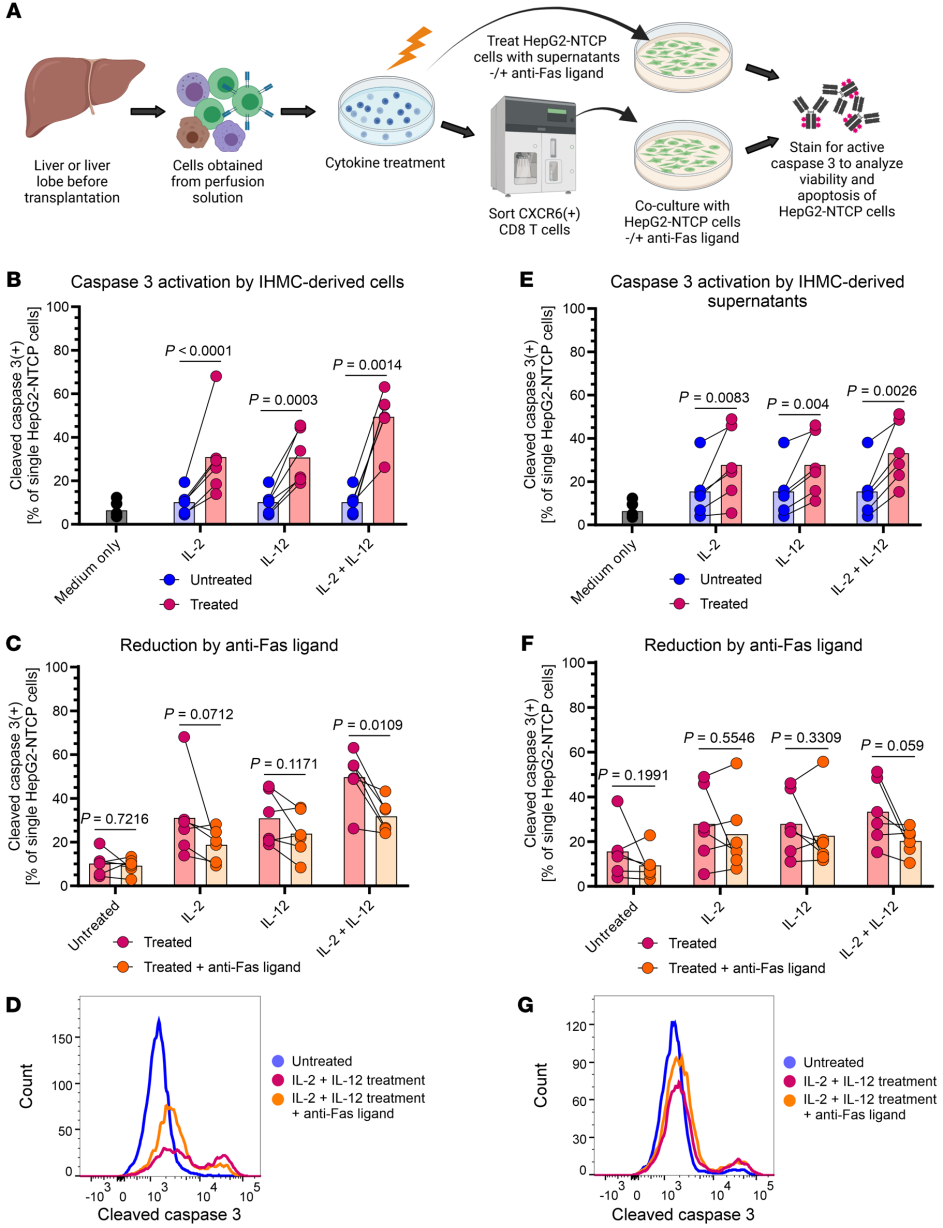
IHMC-derived CD8^+^CXCR6^+^ cells corresponding to the hepatotoxic CD8^+^ T cell population can induce apoptosis in a hepatoma cell line. (**A**) Experimental setup: IHMCs from 6 living liver donors were treated with indicated cytokines to induce the population corresponding to hepatotoxic CD8^+^ T cells. Cells were sorted to obtain the CD8^+^CXCR6^+^ subpopulation and cocultured with HepG2-NTCP cells for 24 hours. In parallel, HepG2-NTCP cells were cultivated in IHMC-derived supernatants. The potential of cells or supernatants to induce apoptosis in HepG2-NTCP cells was evaluated by quantifying active caspase-3 using flow cytometry. In addition, we tested to determine whether a neutralizing anti-FasL antibody could inhibit induction of apoptosis. (**B**) Active caspase-3 in HepG2-NTCP cells that were cocultured with IHMC-derived CD8^+^CXCR6^+^ cells. Circles indicate individual donors; bars indicate mean values. Medium only indicates HepG2-NTCP cells without cocultivation. (**C**) Active caspase-3 in HepG2-NTCP cells after coculture with and without FasL blockade. (**D**) Histogram from 1 representative donor. (**E**) Active caspase-3 in HepG2-NTCP cells that were treated with IHMC-derived supernatants. (**F**) Active caspase-3 in HepG2-NTCP cells after exposure to IHMC-derived supernatants with and without FasL blockade. (**G**) Histogram from 1 representative donor after exposure to IHMC-derived supernatant. Two-tailed, ratio-paired *t* test was used to determine statistical significance.

We next aimed to determine whether the activation profile defining hepatotoxic CD8^+^ T cells can induce nonspecific liver damage through killing of hepatocytes. We used HepG2-NTCP human hepatoma cells as models for hepatocytes. HepG2-NTCP cells were not infected with HBV and not HLA matched with IHMC donors to investigate nonspecific killing independently of HBV antigen presentation (Figure 6). CD8^+^ T cells not treated with cytokines served as control for background lysis that may occur through allogeneic reactivity.

*CXCR6* was the one marker that, out of all 24,469 genes in our scRNA-Seq analysis, correlated best with *FASLG* positivity in each individual hepatotoxic CD8^+^ T cell (not shown). After cytokine stimulation for 24 hours, we sorted CXCR6^+^CD8^+^ T cells to enrich for the CD8^+^ T cell population containing hepatotoxic CD8^+^ T cells. We cocultured sorted cells with HepG2-NTCP cells for 24 hours. In parallel, CD8^+^ T cells were preincubated with a neutralizing anti-FasL antibody before cocultivation. Cleaved (active) caspase-3 was quantified in HepG2-NTCP cells to measure killing (Figure 6A). HepG2-NTCP cells showed a background of 6.5% active caspase-3 positivity. This increased to 10.2% when hepatoma cells were cocultured with CD8^+^CXCR6^+^ cells derived from untreated IHMCs (Figure 6B). IL-2 or IL-12 treatment of IHMCs led to 31.0% and 30.8% of HepG2-NTCP cells with active caspase-3, respectively. When IHMCs were treated with IL-2 plus IL-12, sorted CD8^+^CXCR6^+^ cells induced caspase-3 activation in 49.5% of HepG2-NTCP cells (4.9-fold increase) (Figure 6B). These data show that CD8^+^ T cells with a hepatotoxic CD8^+^ T cell phenotype, activated by IL-2 plus IL-12, mediated enhanced killing of HepG2-NTCP cells.

To determine whether hepatotoxic CD8^+^ T cell–induced hepatoma cell killing was dependent on Fas/FasL, we tested to determine whether FasL blockade could inhibit the apoptosis-inducing effect (Figure 6, C and D). There was no significant difference between cells derived from untreated IHMCs with or without addition of anti-FasL prior to coculture. The same held true for cells derived from IHMCs treated with either IL-2 or IL-12 alone, although FasL blocking had an effect approaching significance. However, when cells were activated with IL-2 plus IL-12, their capacity to kill HepG2-NTCP cells was significantly reduced by blocking FasL (Figure 6C). Blocking FasL reduced HepG2-NTCP killing to 31.7%. This means that about half of the apoptosis-inducing effect of IL-2 plus IL-12–activated IHMC-derived CXCR6^+^CD8^+^ T cells was abolished through FasL blockade when adjusted by subtracting killing observed in untreated cells. Representative histograms (Figure 6D) show a clear decrease in active caspase-3 staining after blocking FasL. This indicates that the Fas/FasL pathway is a key mechanism associated with nonspecific, contact-dependent HepG2-NTCP killing by hepatotoxic CD8^+^ T cells.

We also tested to determine whether we could induce hepatotoxic CD8^+^ T cells in matching PBMCs from IHMC donors (Supplemental Figure 7). The CXCR6^+^CD8^+^ T cell population in PBMCs was small, and IL-2 plus IL-12 exposure did not increase CXCR6 expression (Supplemental Figure 7A). IL-2 plus IL-12 treatment significantly increased IFN-γ, but not FasL expression in CXCR6^+^CD8^+^ T cells (Supplemental Figure 7A), and it did not change the major T cell phenotypes, as analyzed by our multicolor flow cytometry panel (Supplemental Figure 7B). In functional experiments, PBMC-derived CXCR6^+^CD8^+^ T cells activated with IL-2 plus IL-12 displayed only modest killing, with activation of caspase-3 in 13.5% of HepG2-NTCP cells. This effect was lower than the activation induced by IHMC-derived hepatotoxic CD8^+^ T cells, and it was not statistically significant (Supplemental Figure 7C). These data further support the apparent nonresponse of peripheral CD8^+^ T cells.

To assess whether soluble factors also mediated cytotoxicity toward HepG2-NTCP cells, we treated HepG2-NTCP cells with supernatants collected from IHMCs after 24 hours of activation with IL-2 plus IL-12. Supernatants had a less pronounced effect on HepG2-NTCP killing, but still induced caspase-3 activity (Figure 6, E–G). The highest effect was found for supernatants from IL-2 plus IL-12–treated IHMCs, which increased the proportion of active caspase-3^+^ cells 2.1-fold. Antagonism with anti-FasL slightly decreased killing, but not significantly (Figure 6, F and G). This suggests that contact-dependent killing is the primary mechanism, but an additional, contact-independent nonspecific killing mechanism may also be induced by IL-2 plus IL-12 treatment.

We also assessed the contribution of cytotoxic granule–mediated killing by activated hepatotoxic CD8^+^ T cells. Granzyme B (*GZMB*), the primary effector of cytotoxic granules, showed low expression in hepatotoxic CD8^+^ T cells from liver FNAs based on scRNA-Seq data (see Figure 1B), and granzyme B was not significantly changed in IHMCs at the protein level when activated with IL-2 plus IL-12 (Supplemental Figure 8A). After coculture of activated and sorted IHMC-derived hepatotoxic CD8^+^ T cells with HepG2-NTCP cells, CD107a, a marker of degranulation, was not significantly changed on the surface of hepatotoxic CD8^+^ T cells compared with nonactivated cells (2-sided, paired *t* test: *P* = 0.2410), with less than 3% positive cells (Supplemental Figure 8, C and D). When clustering the cells according to markers measured by flow cytometry, CD107a^+^CD8^+^ T cells formed a small cluster with a phenotype distinct from that of hepatotoxic CD8^+^ T cells (Supplemental Figure 8E).

Finally, to exclude enhanced allogeneic killing after IL-2 and IL-12 exposure, we tested the effect of an HLA class I blocking antibody. When cocultivating IHMC-derived hepatotoxic CD8^+^ T cells and HepG2-NTCP cells, we added antibodies to block FasL, HLA class I, or FasL plus HLA class I (Supplemental Figure 9). Compared with no blockade (active caspase-3^+^ HepG2 NTCP cells: 49.8%), HLA blockade reduced apoptosis induction in a limited manner (to 38.3%; *P* = 0.0861), which was weaker than the reduction through FasL blockade (decreased to 28.2%; *P* = 0.0139). When blocking both HLA class I and FasL, caspase-3 activation in HepG2-NTCP cells was reduced to 14.2%. This indicates that HLA-mediated and FasL-mediated killing are 2 separate mechanisms, and it confirms our observation that cell-to-cell contact is required for FasL-mediated killing, which is facilitated through HLA-dependent interaction.

In conclusion, hepatotoxic CD8^+^ T cells derived from IHMCs, but not PBMCs, were induced by treatment with IL-2 and IL-12. Coculture of HepG2-NTCP cells with hepatotoxic CD8^+^ T cells substantially activated apoptosis pathways in HepG2-NTCP cells, which could be inhibited by antagonizing membrane-bound FasL. These data demonstrate a pathway for contact-dependent killing by hepatotoxic CD8^+^ T cells, which can promote liver damage in a nonspecific manner.

## Discussion

Liver FNAs revolutionized our ability to investigate tissue-specific mechanisms of immunity and pathogenesis because the minimal risk allows for sampling on time scales consistent with antiviral effects of therapy. We took advantage of liver FNAs to longitudinally quantify dynamic changes in the activation of individual liver immune cells, as initially elevated ALT levels normalized under antiviral therapy. scRNA-Seq of liver FNAs provided high-resolution data for defining a distinct, activated, liver-resident, polyclonal CD8^+^ T cell population in CHB patients that was capable of nonspecific cytotoxicity through a FasL-dependent pathway. Furthermore, we show that IFN-γ, which induces chemokines associated with immune cell infiltration, can be induced in the CXCR6^+^CD8^+^ T cells through nonspecific activation. This is an important observation, since CHB pathogenesis in animal models relied on IFN-γ produced from functional HBV-specific T cells. Definition of a specific CD8^+^ T cell population primarily responsible for liver damage substantially advances the understanding of pathogenic processes in CHB patients.

By conventional understanding, the hepatotoxic CD8^+^ T cell cluster displayed a paradoxical phenotype, expressing a broad profile of activation marker, chemokines, and cytokines while also expressing the highest levels of immune checkpoint receptors, such as *PDCD1*, *LAG3*, *TIM3*, and *TIGIT*. However, elevated *PDCD1* expression was transient in the scRNA-Seq data, suggesting that it is a marker of activation rather than exhaustion. This is consistent with previous findings, in which PD-1 positively correlated with ALT levels (32–34), was highly expressed on IL-2–producing tissue-resident CD8^+^ T cells (26, 35), and was associated with non-HBV models of chronic inflammation, such as juvenile idiopathic arthritis or polyomavirus encephalitis (36–38). Similarly, a recent study found PD-1 upregulation associated with nonspecific activation of bystander CD8^+^ T cells in chronic hepatitis D (7). These data contrast with what is known of PD-1 expression on HBV-specific CD8^+^ T cells, which, in that context, is a known exhaustion marker (22, 33, 39). Given the size of the hepatotoxic CD8^+^ T cell population, its highly activated profile at baseline, when there is least viral control, and the ability to induce these cells from uninfected healthy livers, it is exceedingly unlikely that these cells represent the HBV-specific T cell response in the CHB patient liver. The high expression of immune checkpoints such as PD-1 in the hepatotoxic CD8^+^ T cell population did not impede CD8^+^ T cell functionality and, as has been demonstrated, expression of these checkpoint inhibitors cannot always be interpreted as CD8^+^ T cell exhaustion in scRNA-Seq data. This has implications for immunotherapy in CHB patients, in whom modulating the liver environment to overcome immune checkpoints may be more effective and safer than systemic administration of checkpoint inhibitor therapy, which can lead to immune-related adverse events.

Features of the hepatotoxic CD8^+^ T cell population in CHB patients are in line with those of other liver diseases; for example, CXCR6^+^CD8^+^ T cells with both high activation levels and immune checkpoint expression were linked to liver damage in nonalcoholic steatohepatitis (40) and to hepatocellular carcinoma (41). With induction through IL-2 plus IL-12, our data suggest a potentially shared mechanism in the liver. Tissue-resident CD8^+^ T cells, which are distinct from our hepatotoxic CD8^+^ T cells, can produce IL-2 in the liver (35). The observation that IL-12 was derived from B cells was unexpected. However, human B cells have the demonstrated capacity to produce IL-12 and play a role in fulminant hepatitis, in which severe inflammation leads to liver failure (42–44).

Mediating clinically relevant liver damage, reflected by elevated serum ALT, requires a sufficient number of activated CD8^+^ T cells to kill hepatocytes and/or the ability of those cells to serially kill multiple hepatocytes. The Fas/FasL system has been associated with liver damage in mouse models and primary human hepatocytes from CHB patients (45–48). Membrane-bound FasL binds to Fas on target cells and induces proapoptotic pathways. However, the soluble form may not induce apoptosis and may even protect target cells through competition for the Fas receptor (49–51). In accordance with the function of membrane-bound versus soluble FasL, we found that strong induction of FasL-mediated apoptosis required a contact-dependent mechanism, while supernatants displayed no significant FasL-dependent killing of HepG2-NTCP cells. CD8^+^ T cells constituted the largest immune population in the liver, and hepatotoxic CD8^+^ T cells were the largest cluster of CD8^+^ T cells, suggesting they are sufficiently abundant to drive liver damage. In addition, our data suggest hepatotoxic CD8^+^ T cells can mediate serial killing of hepatoma cells. We were able to induce FasL expression on 12.9% of CXCR6^+^CD8^+^ T cells, which led to killing 50% of hepatoma cells, indicating each cell has the potential to kill multiple (in our model, 3–4, over 24 hours) hepatocytes.

It is noteworthy that by week 12 of therapy, the majority of ALT decline had occurred, but we observed only modest changes in gene expression. Hepatotoxic CD8^+^ T cells were the only CD8^+^ T cell population with a substantial number of downregulated genes during this window, suggesting they were primarily responsible for liver damage at baseline. However, even in hepatotoxic CD8^+^ T cells, FasL expression had only decreased 1.2-fold by week 12, with distinctly more changes in gene expression by week 24. We interpret this discrepancy as the activation of intrahepatic immunity lagging behind the clinical marker of liver damage, ALT decline. Consistent with the discordant kinetics in TAF-treated HBV patients, acute HCV infection in chimpanzees showed upregulation of key inflammatory genes weeks to months before ALT elevation, whereas hepatocyte necrosis coincided with ALT elevation (52). Our data suggest prolonged intrahepatic immune activation as ALT approached normal values. This is important because ALT is not an ideal marker of intrahepatic immune activity (53) and such a delay could have additional implications for using ALT as a marker when administering immunotherapies.

FNAs have been used extensively to sample tumors and diverse tissues (54–59). A finer needle is used for liver FNAs compared with core biopsies, reducing risks and allowing for frequent sampling. The trade-off is the loss of tissue architecture, lack of high-quality hepatocytes, and some blood contamination (60). However, liver FNAs can consistently capture the immunodiversity within patients’ livers across longitudinal time points. This will be critical when investigating pathogenesis and mechanisms of action of novel therapeutics currently in development to cure chronic HBV infection (24, 29). Another important advance is that FNA cells could be cryopreserved similarly to standard protocols for PBMCs. Despite freezing less than 100,000 cells/vial, we recovered sufficient viable cells to validate scRNA-Seq data by flow cytometry. This opens the window for analyzing single-cell data and returning to cryopreserved samples for validation without enrolling new patients.

Our study combined longitudinal liver FNA sampling with state-of-the-art technology to investigate immunological mechanisms of liver pathogenesis in an investigator-initiated clinical trial. This approach addressed key questions, so far, to our knowledge, only investigated in animal models, to define innate triggers that cause bystander activation of a defined CD8^+^ T cell population capable of TCR-independent hepatocyte killing. Given that liver damage is the primary driver of disease progression, identifying sources of liver damage provides important knowledge for understanding pathogenesis in the natural history of CHB and for developing therapeutic interventions. In conclusion, we demonstrate the value of combining longitudinal liver sampling, scRNA-Seq data analysis, and validation using cryopreserved samples, which can serve as an example for future clinical trials.

## Methods

### CHB patients.

Fifteen patients were initially included in this study to analyze blood, PBMCs, and liver FNAs. All patients concluded the study, with samples taken at all planned time points (baseline, week 12, week 24). Of those, 9 patients’ samples were analyzed in this study. Inclusion criteria were CHB (HBsAg^+^ for 6 or more months); age 18 or more years; high-normal or elevated ALT levels, defined as greater than 19 IU/l for women and greater than 30 IU/l for men (with ULN defined as greater than 25 IU/l for women and greater than 35 IU/l for men); HBV DNA greater than 10,000 IU/ml for HBeAg^+^ and greater than 1,000 IU/ml for HBeAg^–^ patients; adequate contraception. An overview of baseline characteristics is given in Supplemental Table 1. At baseline, all included patients had elevated ALT levels above the ULN. Exclusion criteria were antiviral or IFN treatment in the previous 6 months; immunosuppressive treatment in the previous 6 months; treatment with an investigational drug in the previous 3 months; history of decompensated liver cirrhosis; liver transplantation; coinfection with HCV, HDV, or HIV; other significant liver disease (such as alcoholic or drug-related liver disease, autoimmune hepatitis, hemochromatosis, Wilson’s disease or α1 antitrypsin deficiency); estimated glomerular filtration of less than 50 ml/min/1.73m^2^ or significant renal disease; α-fetoprotein greater than 50 ng/ml; pregnancy or breast feeding; other significant medical illness that might interfere with the study (e.g., immunodeficiency syndromes or malignancies); and substance abuse.

Of the 5 patients analyzed by scRNA-Seq, 4 were men and 1 was a woman. Of the 4 patients analyzed for verification of the transcriptomic data on the protein level, 2 were men and 2 were women.

### Human donors of IHMCs.

Eleven healthy living liver donors were included to obtain IHMCs from liver perfusion solutions during the transplantation and corresponding PBMCs. Mean age at the time of donation was 40.7 years (range 30–54 years). Four patients were men, and seven patients were women.

### HepG2-NTCP cells.

The human hepatoma cell line HepG2-NTCP was originally obtained from a White American male adolescent (61) and modified to overexpress the HBV entry receptor sodium taurocholate cotransporting polypeptide (NTCP) (62). HepG2-NTCP cells were provided by Stephan Urban (Heidelberg University, Heidelberg, Germany). Cells were maintained in DMEM (Gibco, Thermo Fisher) supplemented with 10% FBS, 20 mM HEPES buffer, 100 U/ml penicillin, 100 μg/ml streptomycin, 10 ng/ml plasmocin, 2% minimum essential medium (MEM) amino acids (Gibco, Thermo Fisher), 1% MEM nonessential amino acids (Gibco, Thermo Fisher), 1 mM GlutaMAX (Gibco, Thermo Fisher), and 1 mM sodium pyruvate. When cocultured with IHMC-derived cells, AIM-V medium (Gibco, Thermo Fisher) plus 100 μg/ml primocin plus 2% human AB serum was used.

### Study design.

This was an investigator-initiated, open-label phase 4 study at the Toronto Centre for Liver Disease. Patients started therapy with 25 mg/d TAF, which continued for the entire study duration of 48 weeks, and were offered continued therapy after the end of the study. Blood and FNA samples were collected at baseline, week 12, and week 24. Additional blood samples were collected at weeks 36 and 48.

### Analysis of blood markers of HBV infection.

ALT in patients’ blood was quantified using ADVIA (Siemens). HBV DNA was analyzed with AmpliPrep Taqman (Roche). HBsAg and HBeAg were measured by Architect assay (Abbott). These measurements were done by the Laboratory Medicine Program of Toronto General Hospital/University Health Network.

### PBMC isolation.

Blood was collected from CHB patients at the time of FNA collection or from living liver donors just before transplantation. Red blood cells were removed using density gradient centrifugation at 1200*g* for 10 minutes with brake on at 20°C using Lymphoprep and SepMate 50 isolation tubes (Stem Cell Technology). The supernatants containing PBMCs were transferred, and cells were washed and counted. Cells were cryopreserved in Knockout Serum Replacement plus 10% DMSO.

### Liver FNA collection.

Liver FNAs were collected by a specialist. Twenty-five–gauge needles were used for puncture and aspiration of cells. After removal from the patient, the needle was flushed with an additional 0.5 ml of medium to collect remaining cells. A total of 4 liver FNA passes were taken from each patient at each time point. Samples were maintained on ice and immediately processed: the exact volume of each FNA pass was documented. A small fraction of each pass was used to collect OD to obtain a quantitative measure of the blood content (50). For each analysis, we used the 1 or 2 passes with the lowest blood content from the respective time point and patient. Samples were counted and either cryopreserved in Knockout Serum Replacement (Gibco, Thermo Fisher) plus 10% DMSO for future analysis by flow cytometry or prepared for scRNA-Seq. For scRNA-Seq, red blood cells were removed by 5 minutes of incubation with Red Blood Cell Lysis Buffer (BioLegend) and subsequent ×10 dilution with PBS. Cells were washed, counted, and subjected to scRNA-Seq.

### scRNA-Seq on the 10x Genomics platform.

Samples were prepared as outlined by the 10x Genomics Single Cell 5′ Reagent Kit user guide. Briefly, maximum volume was loaded to target capturing of a maximum of 3,000 cells. After droplet generation, cDNA was generated overnight. The next day, cDNA was recovered using Recovery Agent provided by 10x Genomics and subsequently purified using a Silane DynaBead (Thermo Fisher) mix. Purified cDNA was amplified before being purified again using SPRIselect beads (Beckman Coulter). Samples were run neat on a Bioanalyzer (Agilent Technologies) to determine cDNA concentrations. 5′ cDNA libraries were prepared as outlined by the 10x Genomics’ Single Cell 5′ Reagent Kit user guide, with modifications to the PCR cycles based on the calculated cDNA library input. To obtain TCR repertoire profiles from the same input samples, VDJ enrichment was performed with the Chromium Single Cell Human TCR Amplification Kit (10x Genomics). Sequencing libraries were generated with unique sample indices for each sample and quantified.

For sequencing, the molarity of each library was calculated based on library size as measured by the Bioanalyzer (Agilent Technologies) and qPCR amplification data. Samples were pooled and adjusted to 10 nM, then diluted to 2 nM. Each 2 nM pool was denatured. Library pools were further diluted to a final loading concentration of 14 pM, and 150 μl was loaded into each well of an 8-well strip tube and loaded onto a cBot (Illumina) for cluster generation. Samples were sequenced on the HiSeq 2500 (Illumina) system.

Unique molecular identifiers (UMIs) were generated using the CellRanger Pipeline, version 3.1.0 (10x Genomics). Sequencing data were aligned to the GRCh38-HBV reference genome.

### Analysis of scRNA-Seq data.

Cell Ranger–processed filtered feature matrices were analyzed using Seurat, version 3.2.3 (63). Data from individual samples were filtered to preserve only high-quality cells with more than 100 reads and less than 10% mitochondrial DNA content; genes that appeared in less than 3 cells were filtered out. Data were normalized using Bioconductor’s scran with clusters (64) because normalization was shown to be the most influential step in scRNA-Seq analysis pipelines and scran was superior to other normalization methods (65). Data from all samples were then integrated and scaled. Principal component analysis (PCA) dimensionality reduction was performed, and uniform manifold approximation and projection (UMAP) coordinates were calculated. Clusters were identified using the Louvain algorithm with resolution set to 1. Cell types were annotated based on the canonical marker gene expression of each cluster. Whenever the canonical marker gene expression was ambiguous (this was the case for the cluster of proliferating cells), we used gene set enrichment analysis (GSEA) (66, 67) to identify pathways characterizing the cluster: gene rank lists were compiled in Seurat, GSEA was performed with GSEA, version 4.0.3, and enrichment maps were visualized using Cytoscape, version 3.7.2. Further analysis of scRNA-Seq data was done using R packages EnhancedVolcano, version 1.11.3 (68); scRepertoire, version 1.3.1 (69); and NicheNet, version 1.0.0 (70).

### Targeted gene expression scRNA-Seq.

Targeted gene expression kits (Human Immunology Panel, 10x Genomics) were used for target enrichment and resequencing of libraries prepared for single-cell transcriptomic analysis. Experiments were performed according to the manufacturer’s instructions. Briefly, sequencing libraries were quantified using an Agilent Bioanalyzer (High Sensitivity DNA Kit). If less than 300 ng DNA was available, the library was PCR amplified using the Library Amplification Kit (10x Genomics). Libraries from a total cell count of 49,760 were pooled into 3 sets based on input cell counts: low (600–1400 cells/library), medium (1800–2500 cells/library), and high (4200–7700 cells/library). The dilution factors for pooling were calculated using the worksheet provided by the manufacturer. After adding COT DNA and universal blockers, the library pools were dried in a vacuum centrifuge at 45°C (SpeedVac SPD210, Thermo Fisher). Target genes (1,056 immune-related genes) were enriched by hybridizing to gene-specific biotinylated baits bound to streptavidin beads. After washing capture beads, enriched libraries were PCR amplified using the Library Amplification Kit with 10 total cycles and purified using SPRIselect reagent according to the manufacturer’s protocol (Beckman Coulter). The enriched libraries were sequenced on a NovaSeq platform (Illumina) with a sequencing depth of 10,000 read pairs per cell.

Sequencing data were aligned to the GRCh38 reference genome and quantified using Cell Ranger, version 6.0.1 (10x Genomics). Cells were filtered based on the EmptyDrops method (71) implemented in Cell Ranger. After filtering, a total of 39,565 cells remained for downstream analysis. Seurat, version 4.0 (72), was used to normalize cells using the LogNormalize method. PCA dimensionality reduction was performed, and UMAP coordinates were calculated. Clusters were identified using the Louvain algorithm with resolution set to 0.8. Cell types were annotated based on the canonical marker gene expressions of each cluster.

### Flow cytometric analysis of liver FNAs and matched PBMCs.

Preparation of liver FNAs was optimized to minimize cell loss. Longitudinal liver FNAs and matched PBMCs from the same patients were thawed. Dead cells were stained with eFluor 520 (eBioscience). An equal volume of 2× concentrated extracellular antibodies was added. Further information on antibodies used is listed in Supplemental Table 3. Cells were washed and fixed for flow cytometry analysis. Data were analyzed with FlowJo, version 10.7.1.

### Analysis of clusters defined by flow cytometry using flowCore and CATALYST.

Flow cytometry data were further analyzed using R packages flowCore, version 2.5.0 (73), and CATALYST, version 1.17.3 (74). Single, live CD3^+^ lymphocytes were gated in FlowJo, and fcs files were imported into R without truncation for further analysis. Data from FNA or PBMC samples from the same time point were clustered using a 10 × 10 grid and evaluation of 2 through 20 metaclusters. A delta area plot was used to determine the optimal number of clusters. UMAP dimensional reduction was performed on all cells.

### Living liver donor IHMC collection.

IHMCs were isolated from living donor liver transplantation perfusions at Toronto General Hospital. Prior to transplantation, livers were perfused with 1 to 2 L of cold University of Wisconsin (UW) solution (ViaSpan) to remove immune cells from the sinusoids. Perfusates were collected and centrifuged at 500*g* for 10 minutes at 4°C to concentrate cells. Concentrated perfusates were resuspended in HBSS, and red blood cells were removed by layering cell suspension onto Lymphoprep, followed by centrifugation for 30 minutes at 400*g* at 19°C with no brake. Supernatants containing IHMCs were washed, counted, and cryopreserved.

### Cytokine stimulation of IHMCs and PBMCs and flow cytometry analysis.

IHMCs or PBMCs were maintained in AIM-V medium plus 100 μg/ml primocin plus 2% human AB serum. After thawing, they were stained with CXCR6_BV421. Cells were adjusted to a concentration of 1.5 million cells/ml for cultivation and cytokine treatment. Indicated cytokines were used at the following concentrations: IL-2 (GoldBio) at 100 IU/ml; and IL-12p70 (BioLegend) at 25 ng/ml. Additional cytokines were used in exploratory experiments: 25 ng/ml IL-4; 25 ng/ml IL-10; 25 ng/ml IL-15; 25 ng/ml IL-21; 25 ng/ml IL-27; 10 ng/ml TGF-β; 100 ng/ml TNF-α; 100 IU/ml IFN-α; and 100 IU/ml IFN-γ. Further information on the cytokines used is listed in Supplemental Table 3. For positive control of degranulation assay, IHMCs were treated with 10 ng/ml PMA plus 1 μg/ml ionomycin. For analysis of intracellular markers, cytokine treatment was performed in the presence of 1 μg/ml brefeldin A. IHMCs or PBMCs were treated for 24 hours before supernatants were collected for further HepG2-NTCP treatment. Cells were collected for flow cytometry analysis or for sorting and subsequent cocultivation with HepG2-NTCP cells.

For flow cytometry analysis, eFluor 506 (eBioscience) in PBS was used for staining of dead cells. Cells were then incubated with extracellular antibodies. We performed CXCR6 staining both before and after cultivation to capture as many CXCR6^+^ cells as possible. Cells were permeabilized, followed by incubation with intracellular antibodies. Suppliers of all antibodies are listed in Supplemental Table 3. Cells were washed and fixed for analysis.

For flow cytometry analysis using the multicolor panel with markers defining the hepatotoxic CD8^+^ T cell population, IHMCs or PBMCs were thawed, stained with CXCR6_BV421, and treated for 24 hours as described above. Dead cells were then stained with eFluor 520 (eBioscience). Subsequently, cells were stained with extracellular antibodies listed under *Flow cytometric analysis of liver FNAs and matched PBMCs*. Cells were washed and fixed for flow cytometry analysis. Data were analyzed with FlowJo, version 10.7.1, and with flowCore, version 2.5.0, and CATALYST, version 1.17.3, as described under *Analysis of clusters defined by flow cytometry using flowCore and CATALYST*.

### Sorting of IHMCs or PBMCs to select CD8^+^CXCR6^+^ cells.

After cytokine stimulation for 24 hours, IHMCs or PBMCs were sorted prior to cocultivation with HepG2-NTCP cells. Mononuclear cells were kept on ice during preparation and before and after sorting unless indicated otherwise. They were stained with viability dye eFluor520 prior to antibody staining. Cells were washed and resuspended in MACS buffer. We used a Sony SH800 BRV cell sorter to select live, CD8^+^CXCR6^+^ cells. Depending on the setup, sorted cells were or were not preincubated with 10 μg/ml neutralizing anti-FasL antibody (BioLegend) and/or 3 μg/ml neutralizing anti-HLA class I antibody (BioLegend) for 30 minutes at 37°C. Cells with and without blocking antibodies were then added to the HepG2-NTCP cells for 24 hours of cocultivation.

### Positive control for HLA class I blocking.

HBsAg-specific CD8^+^ T cells were generated as previously described (75, 76). HepG2-NTCP cells were seeded at 50,000 cells/well in a 96-well plate. After 24 hours, they were pulsed with 10 nM HBs-derived peptide (HBsAg183-191, amino acids FLLTRILTI) for 30 minutes to induce HBsAg peptide presentation. Cells were washed and incubated with or without 3 μg/ml anti-HLA class I antibody for 30 minutes before addition of 100,000 HBsAg-specific CD8^+^ T cells. Cocultivation was performed for 16 hours in the presence of 1 μg/ml brefeldin A before flow cytometric analysis of CD8^+^ T cells for viability (eFluor 506), CD8, and IFN-γ.

### HepG2-NTCP killing assay.

HepG2-NTCP cells were seeded at 50,000 cells/well in a 96-well plate one day prior to coculture with IHMC-derived CD8^+^ T cells. Sorted CXCR6^+^CD8^+^ T cells were then added to the HepG2-NTCP cells at a 1:1 ratio and cultured for 24 hours. In parallel, HepG2-NTCP cells were cultured in the IHMC-derived supernatants (50 μl/well) for 24 hours. Adherent HepG2-NTCP cells were detached from the cell culture plates. Dead cells were stained using eFluor 506 (eBioscience), and cells were fixed. To permeabilize cellular membranes, including mitochondrial and nuclear membranes, cells were incubated with BD Phosflow Perm Buffer III (BD Biosciences) containing 87.86% methanol. They were washed before incubation with the cleaved caspase-3 AF647 antibody. Cells were washed again before flow cytometry analysis. Cells were analyzed using FlowJo, version 10.7.1.

### Statistics.

Statistical analysis was done using GraphPad Prism 8 and R, version 4.1.0. Statistical tests used and numbers of replicates are indicated in the Results section and figure legends. *P* < 0.05 was considered statistically significant.

### Data availability.

scRNA-Seq data have been deposited in the NCBI’s Gene Expression Omnibus database (GSE216314).

### Study approval.

The study including all hepatitis B patients reported in this work was approved by the University Health Network Research Ethics Board (CAPCR ID 18-5748). Written, informed consent was obtained from all subjects prior to inclusion in the study. The study number is CO-US-320-4667. The study including all IHMC donors reported in this work was approved by the University Health Network Research Ethics Board (CAPCR ID 14-7425). Written, informed consent was obtained from all subjects prior to inclusion in the study.

## Author contributions

JJF, HLAJ, and AJG conceived the project. SN, DM, AK, SCK, and CH curated the data. SN performed formal analysis. AJG acquired funding. ISN, DM, AK, JDSV, SCK, AM, and AP performed experiments. Methodology was designed by SN, DM, JJF, HLAJ, and AJG. CH, JJF, HLAJ, and AJG performed project administration. IM, JJF, SF, HLAJ, and AJG provided resources. JJF, JJW, HLAJ, and AJG supervised the project. Visualizations for the manuscript were done by SN. SN and AJG wrote the original draft. SN, AK, SCK, JJF, SF, DC, JJW, AG, HLAJ, and AJG reviewed and edited the manuscript.

## Supplementary Material

Supplemental data

## Figures and Tables

**Table 1:**
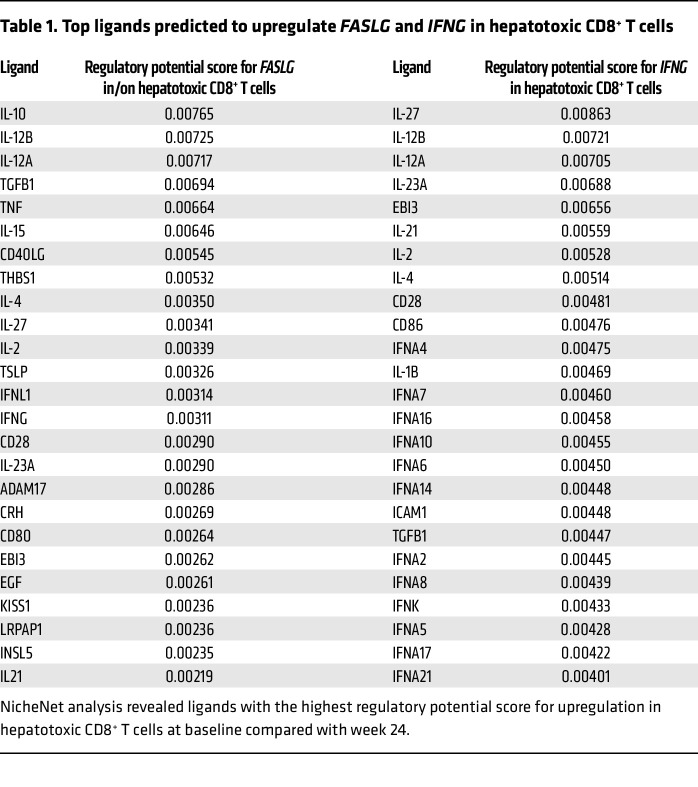
Top ligands predicted to upregulate *FASLG* and *IFNG* in hepatotoxic CD8^+^ T cells

## Version 1 01/03/2023

Electronic publication
