# Supplementary material for: Longitudinal liver sampling in patients with chronic hepatitis B starting antiviral therapy reveals hepatotoxic CD8+ T cells
Source: J Clin Invest. 2023 Jan 3;133(1):e158903. doi: 10.1172/JCI158903 (PMC9797343; doi:10.1172/JCI158903)
Supplement: Supplemental data [file jci-133-158903-s061.pdf]

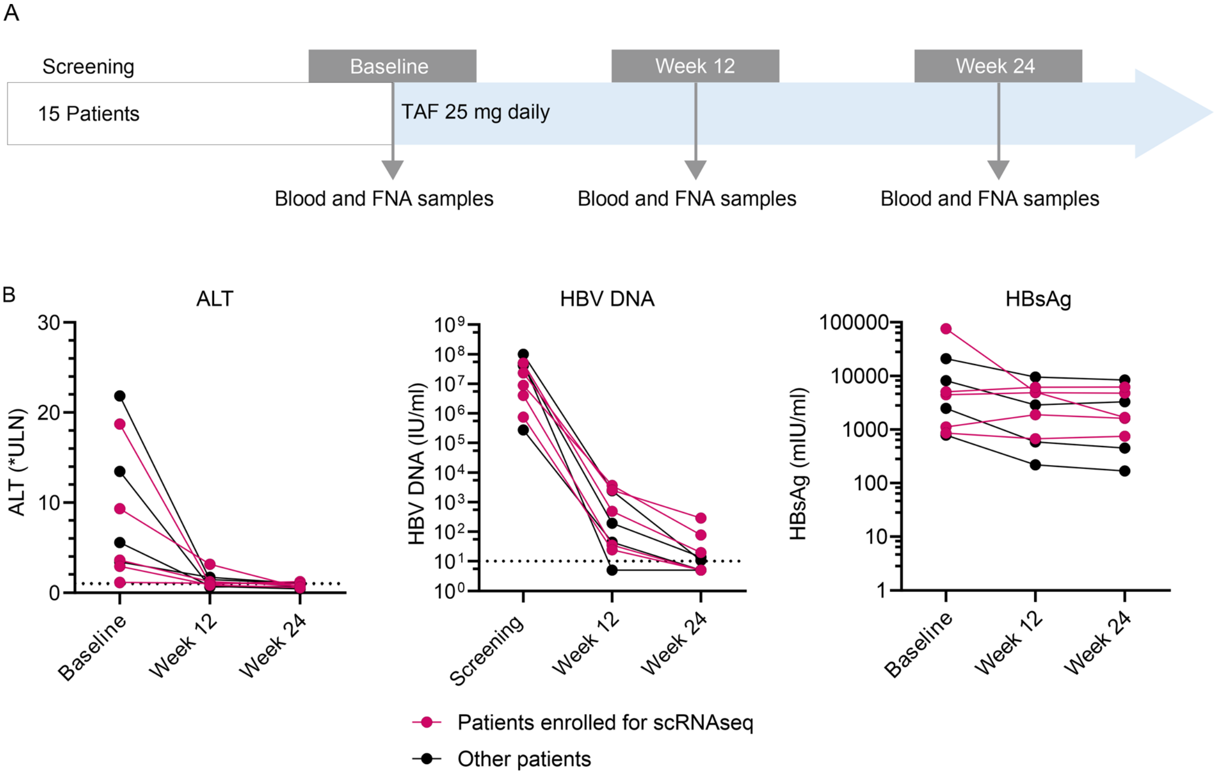

Figure S1. Study design and blood markers of HBV infection under tenofovir alafenamide (TAF) therapy. (A) 9 chronic hepatitis B (CHB) patients with elevated alanine aminotransferase (ALT) levels started nucleoside analogue therapy with TAF 25 mg/d. At baseline and after 12 and 24 weeks of therapy, blood and liver fine-needle aspirates (FNAs) were collected. Longitudinal FNAs from 5 patients were subjected to single-cell RNA sequencing (scRNAseq). (B) ALT (displayed as fold-change of upper limit of normal), HBV DNA and HBsAg levels in the blood over time. Patients indicated in red are those whose samples were analyzed by scRNAseq.

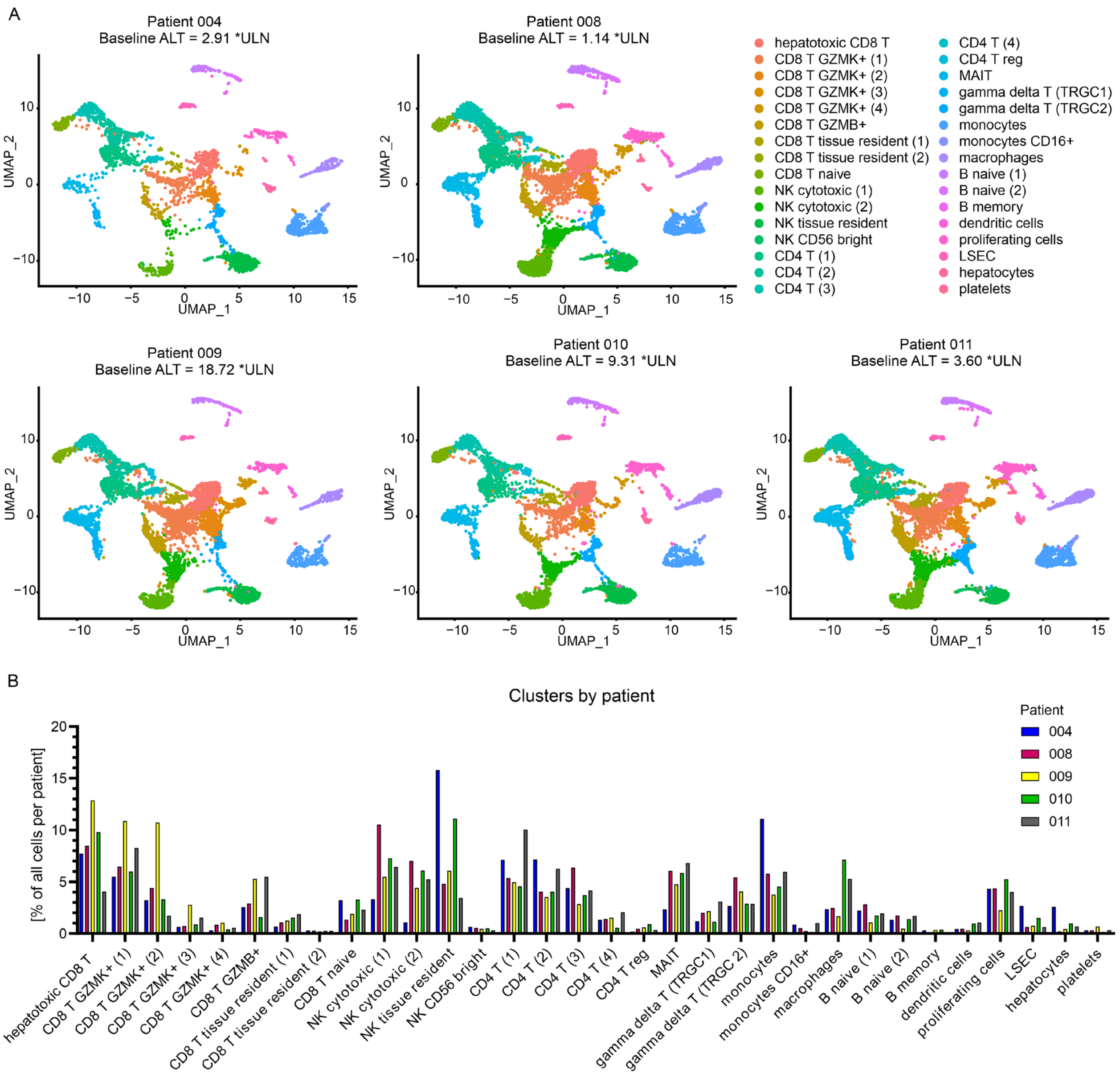

Figure S2. Populations obtained from scRNAseq were equally distributed among all patients. (A) UMAP plots, corresponding to the one in Figure 1A, for each individual patient. (B) Distribution of clusters among all cells, by patient.

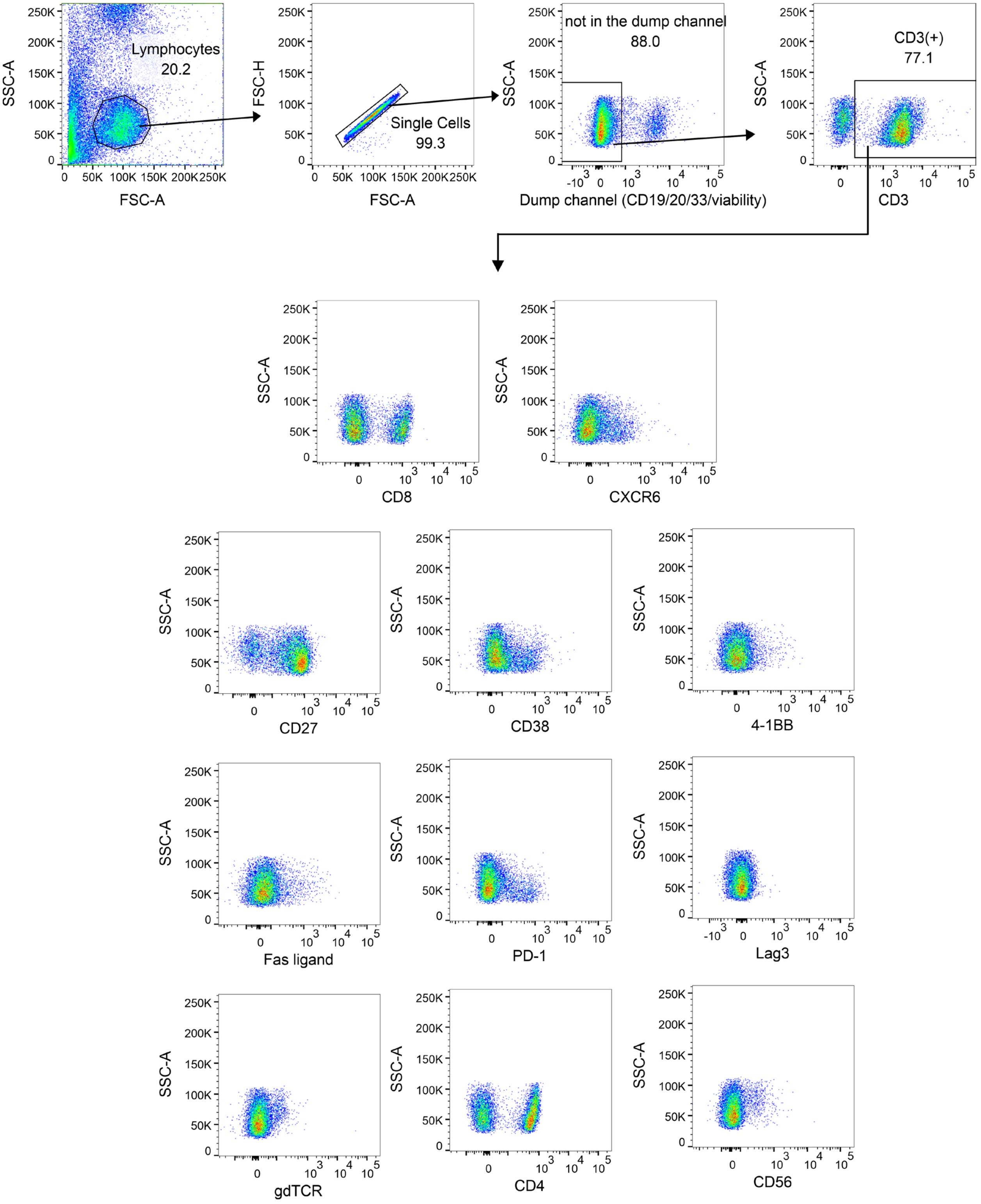

Figure S3. Gating strategy used for clustering of flow cytometry data. Multi-color flow cytometry of one representative FNA baseline sample.

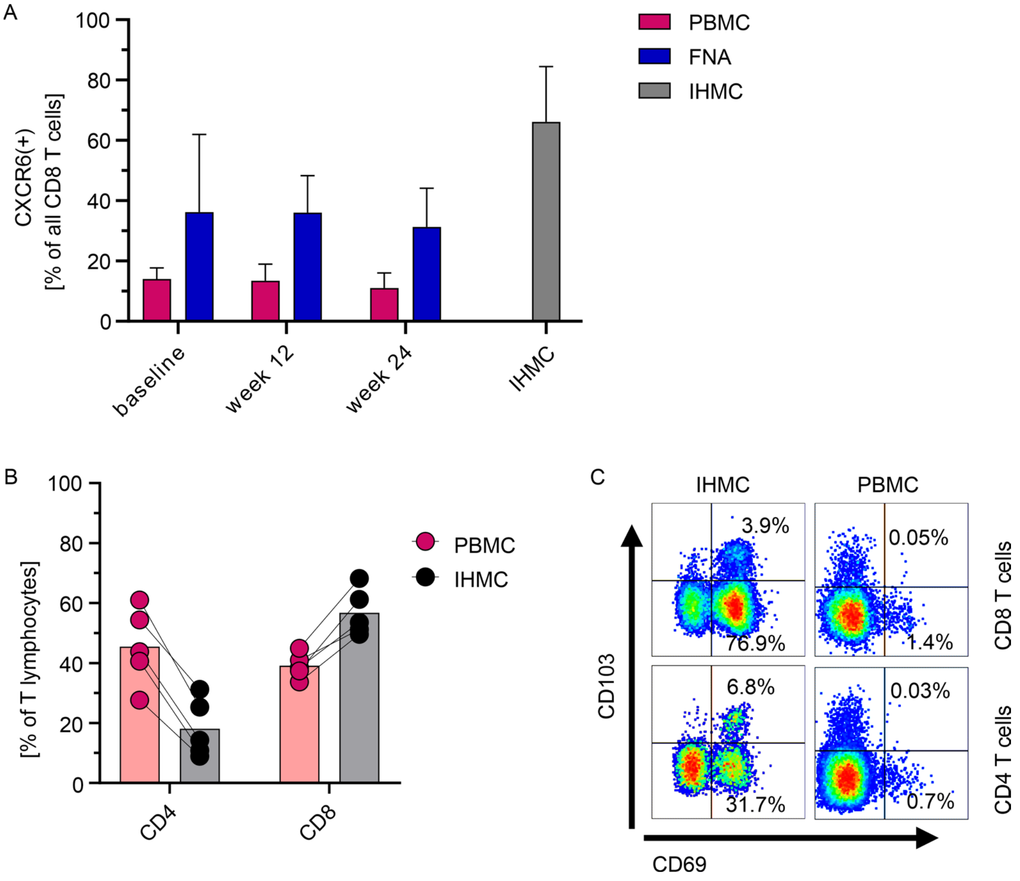

Figure S4. Tissue residency characteristics of intrahepatic lymphocytes, compared to PBMC lymphocytes. (A) Flow cytometry of FNA and matched PBMC of 4 CHB patients before and during TAF therapy; and of 5 living liver donor IHMC. CXCR6 as a marker of tissue residency was quantified in CD8 T cells. Bars display mean values +/- standard deviation. (B) Quantification of CD4 and CD8 T cells in PBMC and matched intrahepatic cells from living liver donors. (C) CD69 and CD103 in CD4 and CD8 T cells derived from PBMC, or matched IHMC, from one representative donor.

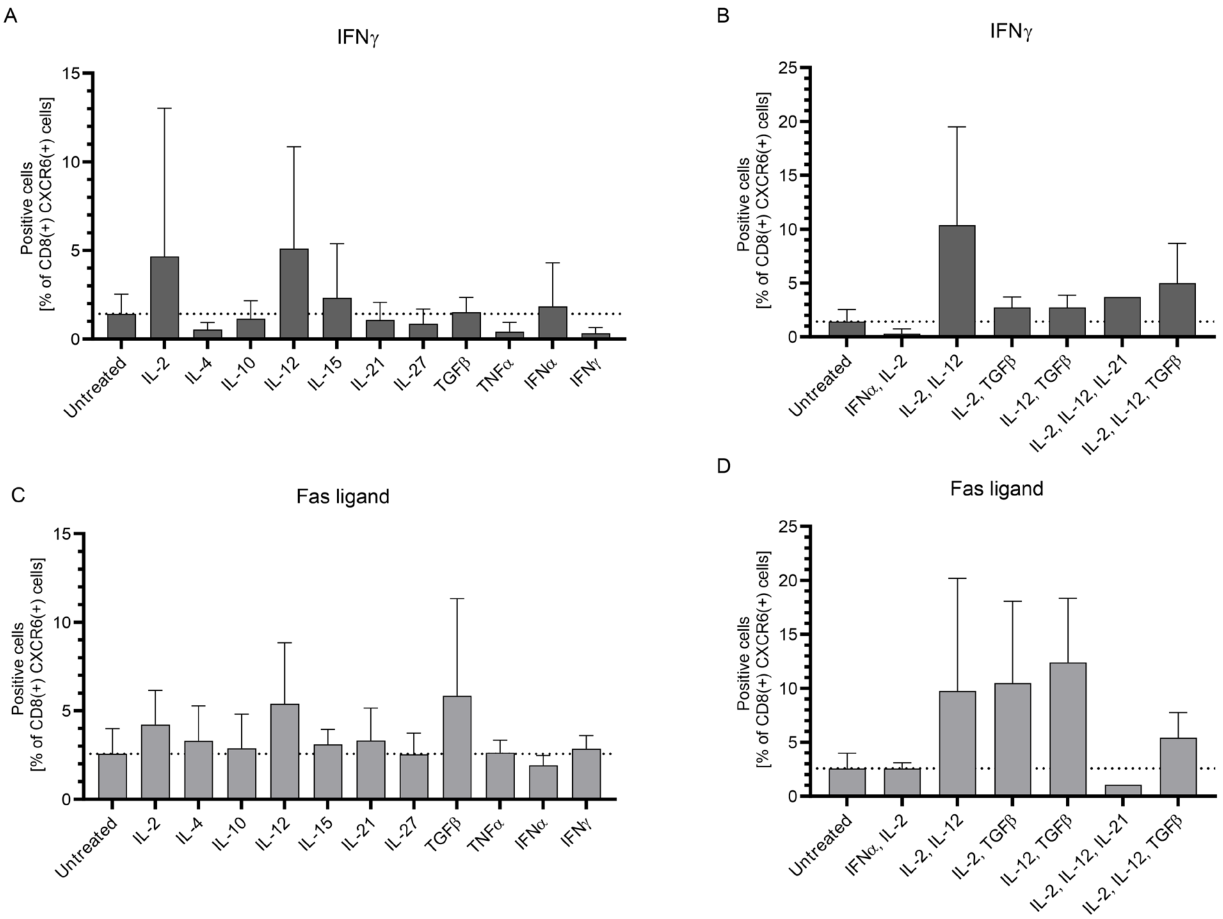

Figure S5. The combination of IL-2 + IL-12 is most effective in inducing key markers of hepatotoxic CD8 T cells in healthy donor IHMC. Cytokines that had been *in silico* predicted to upregulate IFNγ and Fas ligand, key markers of hepatotoxic CD8 T cells in CHB patients at baseline, were assessed with respect to their potential to upregulate those markers in healthy donor IHMC. IHMC from 6 donors were treated for 24 h with the indicated cytokines before IFNγ (A-B) and Fas ligand (C-D) were quantified in CXCR6(+) CD8 T cells by flow cytometry. Bars display mean values +/- standard deviation. (A, C) individual predicted markers, (B, D) selected combinations of 2 and 3 different cytokines.

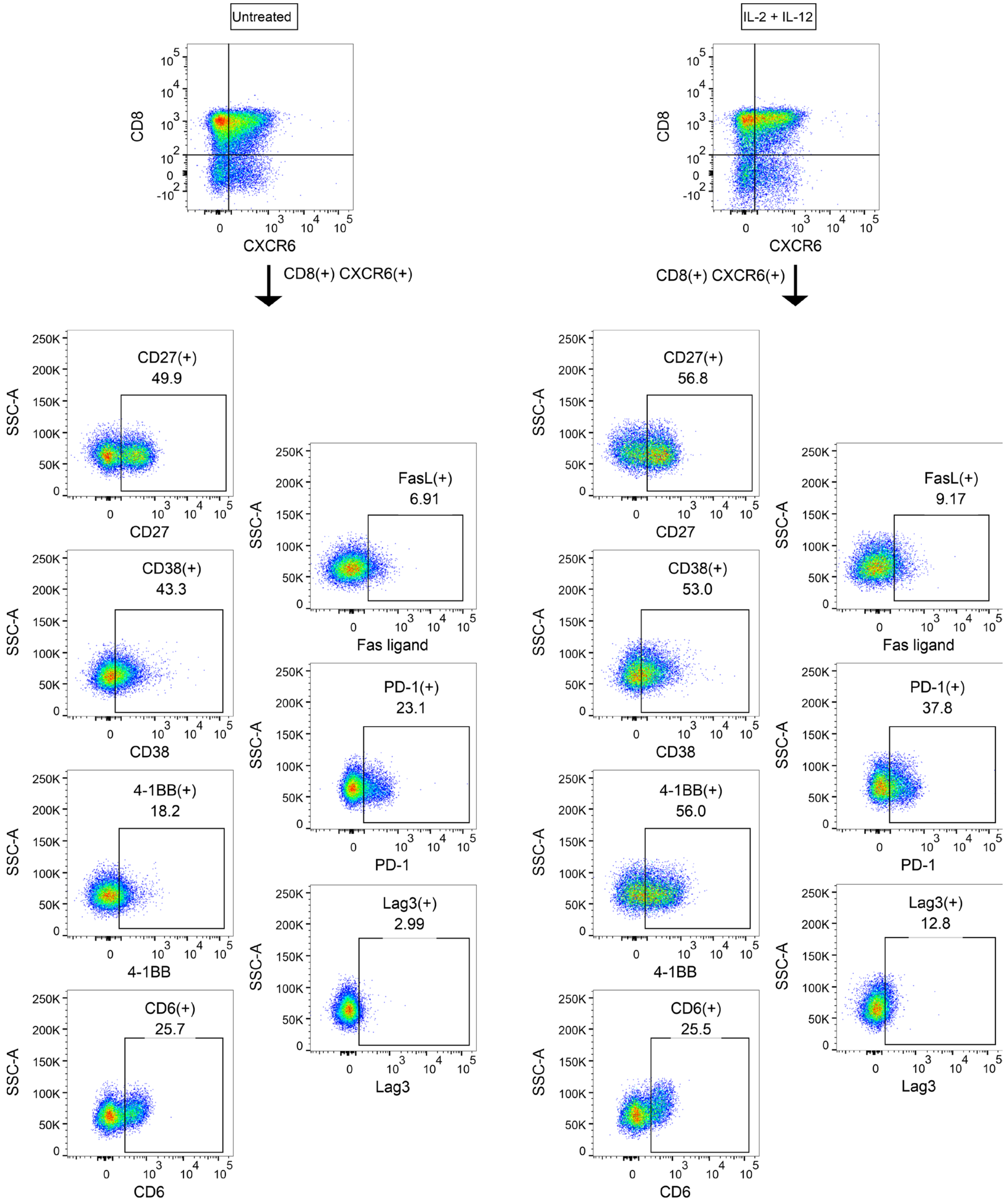

Figure S6. Hepatotoxic CD8 T cell markers in healthy donor-derived IHMC induced by IL-2 and IL-12. Multi-color flow cytometry of one representative donor. Gating:

Lymphocytes → single cells → live cells → CD3(+) → CD8(+) and CXCR6(+) → phenotypic markers that define the hepatotoxic CD8 T population.

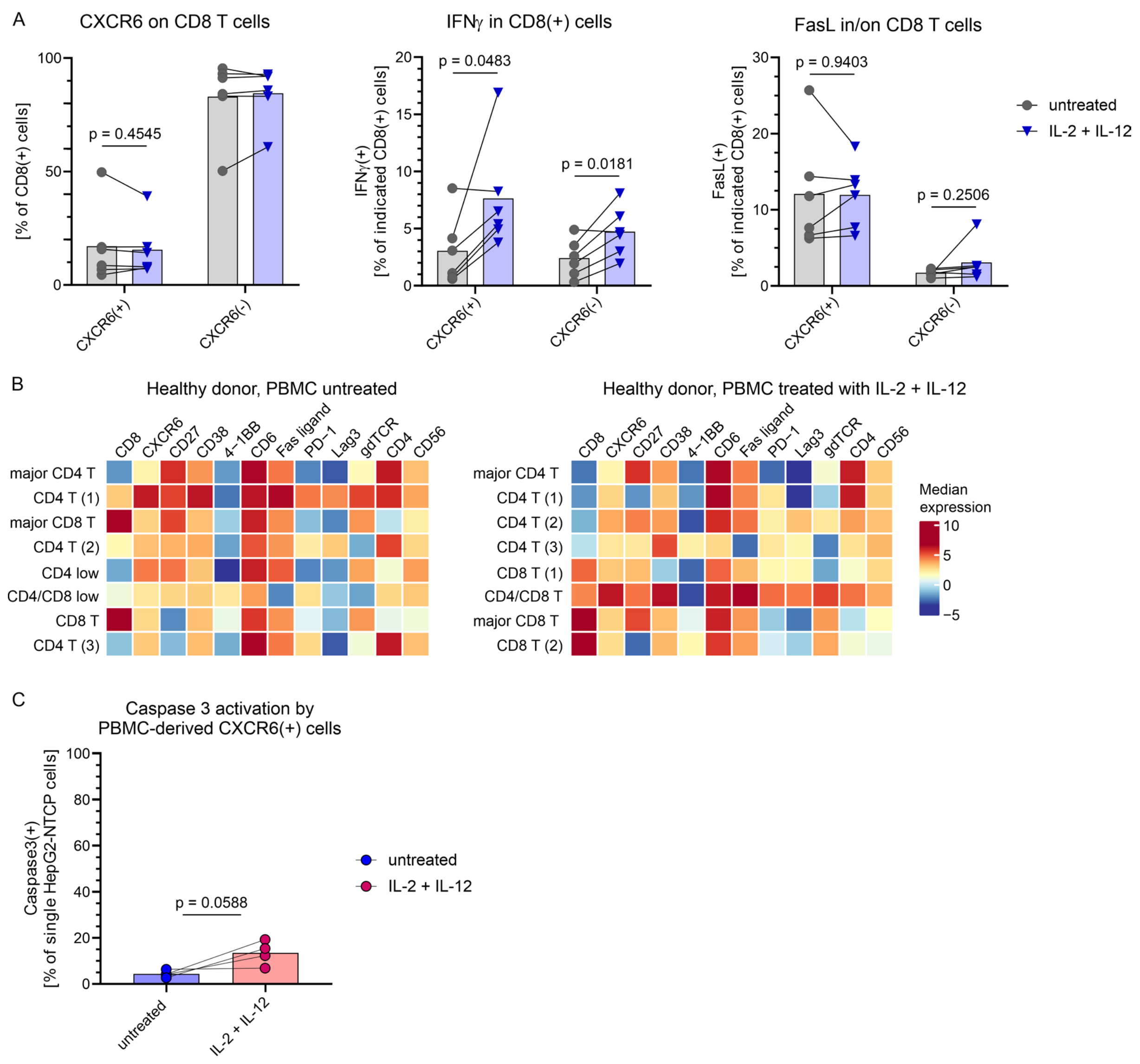

Figure S7. No hepatotoxic CD8 T cell induction through IL-2 + IL-12 in PBMC. (A) PBMC from 6 healthy donors were stimulated with IL-2 and IL-12 before flow cytometric quantification of CXCR6, and quantification of IFNγ and Fas ligand in/on CXCR6(+) and CXCR6(-) CD8 T cells. Two-sided paired t test was used to evaluate significance. (B) Corresponding cells from 5 donors were evaluated by multi-color flow cytometry and clustering of CD3(+) lymphocytes. No cluster of treated cells showed the hepatotoxic CD8 T cell signature. Heatmaps display median expression. (C) For experimental setup, see

Figure 5A. Active caspase 3 in HepG2-NTCP cells that were co-cultivated with PBMC-derived CXCR6(+) CD8 T cells from 4 donors. Significance was assessed with two-sided ratio paired t test.

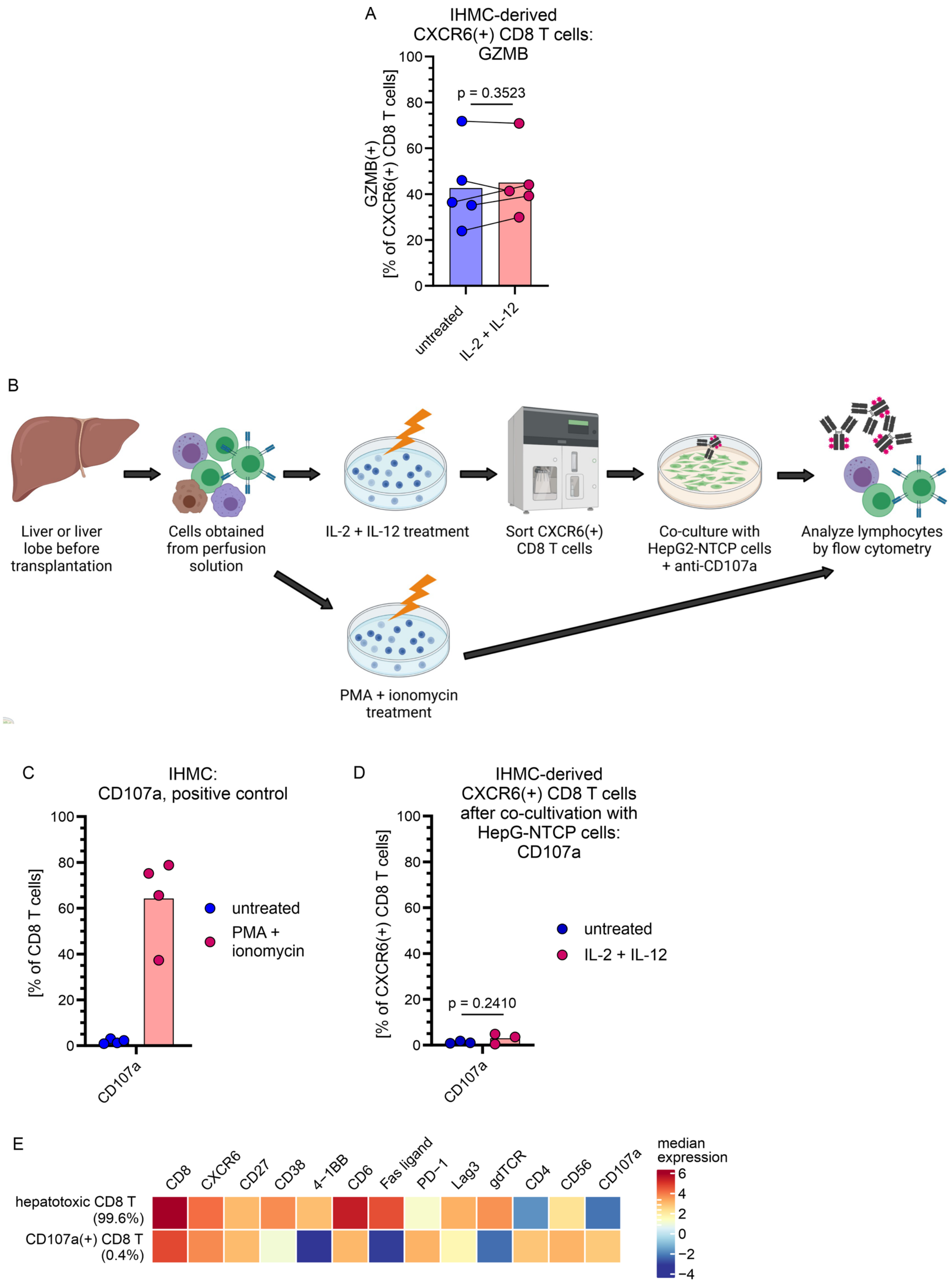

Figure S8. No evidence of degranulation of hepatotoxic CD8 T cells upon activation. (A) IHMC from 5 donors were treated with IL-2 + IL-12 to induce hepatotoxic

CD8 T cells. Intracellular granzyme B was measured by flow cytometry. (B) Experimental setup. IHMC-derived hepatotoxic CD8 T cells from 4 donors were induced as before. CXCR6(+) CD8 T cells were sorted and co-cultivated with HepG2-NTCP cells to induce contact with target cells for 24 h. Lymphocytes were then analyzed by flow cytometry. As a positive control, IHMC were treated with PMA + ionomycin for 24 h, which is known to strongly induce activation and degranulation. (C) Extracellular staining of CD107a as a marker for degranulation of PMA + ionomycin-treated IHMC, or of (D) activated IHMC-derived hepatotoxic CD8 T cells after co-cultivation with HepG2-NTCP cells. Statistical significance was determined by two-sided paired t test. (E) IHMC-derived hepatotoxic CD8 T cells after co-cultivation were analyzed by multi-color flow cytometry. Clustering revealed one dominant cluster with the typical hepatotoxic CD8 T cell signature, and a separate cluster with high expression of CD107a.

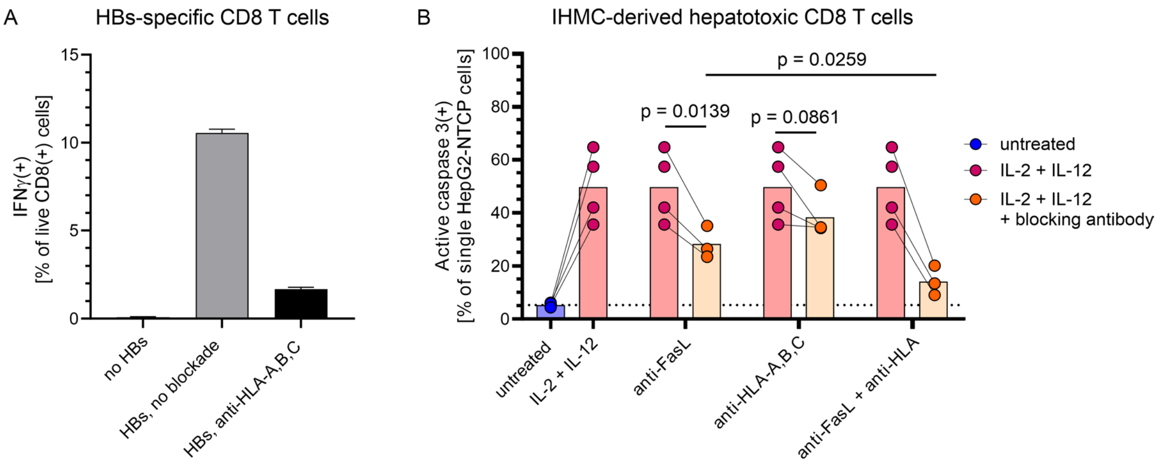

Figure S9. HLA-mediated allogeneic killing and FasL-mediated killing through hepatotoxic CD8 T cells are additive. (A) Positive control for HLA blockade. HepG2-NTCP cells were pre-incubated with HBs183-191 to induce peptide presentation before co-cultivation with HBs-specific CD8 T cells. CD8 T cell activation was evaluated by flow cytometric quantification of IFNγ expression. No peptide presentation (no addition of HBV peptide) lead to no CD8 T cell activation, while HLA blockade during co-cultivation reduced activation 6.3-fold. Bars represent mean values +/- standard deviation of 3 replicates. (B) Reduction of hepatoma cell killing through hepatotoxic CD8 T cells by Fas ligand and HLA blockade. For experimental setup, see Figure 5A. Active caspase 3 in HepG2-NTCP cells co-cultivated with IHMC-derived and IL-2 + IL-12-activated hepatotoxic CD8 T cells from 4 donors, without and with blockade of FasL and/or HLA-A,B,C. Statistical significance was assessed by using two-tailed ratio paired t test.

Table S1. Characteristics of included patients with chronic hepatitis B.

	Mean	Range
Age [years]	45.2	29–64
Male sex [% of all patients]	67%	
ALT at baseline [× ULN]	8.9	1.1–21.8
HBV DNA at screening [IU/ml]	3.09×10^7	2.73×10^5–9.97×10^7
Baseline HBeAg(+) [% of all patients]	44%	

Table S2. Numbers of cells obtained from each patient in each scRNAseq cluster.

patient:	004	008	009	010	011
hepatotoxic CD8 T	278	768	1033	615	450
CD8 T GZMK+ (1)	198	586	873	376	915
CD8 T GZMK+ (2)	116	398	861	208	192
CD8 T GZMK+ (3)	24	67	224	56	170
CD8 T GZMK+ (4)	12	78	85	26	62
CD8 T GZMB+	92	263	426	99	607
CD8 T tissue resident (1)	25	98	100	97	208
CD8 T tissue resident (2)	11	26	17	16	27
CD8 T naïve	116	122	153	206	255
NK cytotoxic (1)	119	954	440	456	714
NK cytotoxic (2)	39	636	354	382	582
NK tissue resident	568	434	487	697	383
NK CD56 bright	24	49	37	32	36
CD4 T (1)	256	485	399	287	1111
CD4 T (2)	257	366	284	253	694
CD4 T (3)	158	577	229	233	463
CD4 T (4)	48	128	124	35	230
CD4 T reg	7	42	48	57	39
MAIT	84	548	382	367	753
gamma delta T (TRGC1)	43	181	175	72	344
gamma delta T (TRGC2)	96	491	327	181	319
monocytes	398	522	302	286	661
monocytes CD16+	31	48	19	10	114
macrophages	85	225	135	447	585
B naïve (1)	80	256	87	110	216
B naïve (2)	48	159	38	88	191
B memory	12	5	29	24	14
dendritic cells	16	43	26	60	118

proliferating cells	156	395	181	330	445
LSEC	96	58	62	95	69
hepatocytes	93	19	36	61	77
platelets	12	29	56	10	37

Supplemental table 3: Key resources

RESOURCE	SOURCE	IDENTIFIER
**Antibodies**		
CD3_BV510, mouse anti-human	BD Biosciences	Cat#563109<br>Clone UCHT1
CD4_BUV395, mouse anti-human	BD Biosciences	Cat#563550<br>Clone SK3
CD6_BUV737, mouse anti-human	BD Biosciences	Cat#749476<br>Clone M-T605
CD8_APC, mouse anti-human	BD Biosciences	Cat#561421<br>Clone RPA-T8
CD8_APC-H7, mouse anti-human	BD Biosciences	Cat#560179<br>Clone SK1
CD19_FITC, mouse anti-human	BioLegend	Cat#302206<br>Clone HIB19
CD20_FITC, mouse anti-human	BioLegend	Cat#302304<br>Clone 2H7
CD27_PE-Cy7, mouse anti-human	BD Biosciences	Cat#560609<br>Clone M-T271
CD33_BB515, mouse anti-human	BD Biosciences	Cat#564588<br>Clone WM53
CD38_BV786, mouse anti-human	BD Biosciences	Cat#563964<br>Clone HIT2
CD56_APC, mouse anti-human	BD Biosciences	Cat#555518<br>Clone B159
4-1BB (CD137)_BV650, mouse anti-human	BD Biosciences	Cat#564092<br>Clone 4B4-1
Fas ligand (CD178)_BV605, mouse anti-human	BD Biosciences	Cat#744099<br>Clone NOK-1
CXCR6 (CD186)_BV421, mouse anti-human	BD Biosciences	Cat#566007<br>Clone 13B 1E5

Lag3 (CD223)_APC-R700, mouse anti-human	BD Biosciences	Cat#565774<br>Clone T47-530
PD-1 (CD279)_BB700, mouse anti-human	BD Biosciences	Cat#566460<br>Clone EH12.1
gdTCR_BUV563, mouse anti-human	BD Biosciences	Cat#748534<br>Clone 11F2
IFNg_APC, mouse anti-human	Biolegend	Cat#506510<br>Clone B27
Granzyme B_PE-Cy7 mouse anti-human	Biolegend	Cat#372214<br>Clone QA16A02
CD107a_PE mouse anti-human	BD Biosciences	Cat#560948<br>Clone H4A3
Cleaved Caspase-3_AF647, rabbit anti-human	Cell Signaling	Cat#9602S<br>Lot 9
viability dye eFluor506	eBiosciences	Cat#65-0866-14
viability dye eFluor520	eBiosciences	Cat#65-0867-14
Fas ligand (CD178) Ultra-LEAF Purified, mouse anti-human	BioLegend	Cat#306415<br>Clone NOK-1
HLA-A,B,C Ultra-LEAF Purified,	Biolegend	Cat#311428<br>Clone W6/32
**Cytokines**		
IL-2	Goldbio	Cat#1110-02-50
IL-4	Goldbio	Cat#1110-04-5
IL-10	Goldbio	Cat#<br>1110-10-2
IL-12p70	BioLegend	Cat#573002
IL-15	Goldbio	Cat#<br>1110-15-10
IL-21	Goldbio	Cat#

		1110-21-10
IL-27	Biolegend	Cat#<br>589202
TGFβ	StemCell	Cat#78067.1
TNFα	Goldbio	Cat#1130-01-10
IFNα	Goldbio	Cat#1160-03-20
IFNγ	Goldbio	Cat#1160-06-20
**Software**		
R versions 3.6.3 – 4.1.0	The R Project	https://www.r-project.org/
Seurat versions 3.2.3 and 4.0	Satija lab	https://satijalab.org/seurat/index.html<br>Stuart et al., 2019<br>Hao et al., 2021
scran	Bioconductor	https://bioconductor.org/packages/release/bioc/html/scran.html<br>Lun et al, 2016
EnhancedVolcano version 1.11.3	Kevin Blighe	https://github.com/kevinblighe/EnhancedVolcano<br>Blighe et al., 2021
scRepertoire	Nick Borcherding	https://github.com/ncborcherding/scRepertoire<br>Borcherding et al., 2020
NicheNet	Robin Browaeys	https://github.com/saeyslab/nichenetr<br>Browaeys et al., 2020
flowCore version 2.5.0	Bioconductor	https://bioconductor.org/packages/release/bioc/html/flowCore.html<br>Ellis et al., 2021
CATALYST version 1.17.3	Bioconductor	https://bioconductor.org/packages/release/bioc/html/CATALYST.html

		Crowell et al., 2021
GraphPad Prism 8	GraphPad	
FlowJo version 10.7.1	FlowJo, LLC	
